# Crossing scales and eras: Correlative multimodal microscopy heritage studies

**DOI:** 10.1111/jmi.70030

**Published:** 2025-09-12

**Authors:** Charles Wood, George Deakin, Atousa Moayedi, Jovana Radulovic

**Affiliations:** ^1^ CoMic Research Group University of Portsmouth Portsmouth UK; ^2^ School of Electrical and Mechanical Engineering University of Portsmouth Portsmouth UK

**Keywords:** artefact analysis, conservation science, correlative multimodal microscopy (CoMic), cultural heritage, electron microscopy, non‐destructive analysis, X‐ray microscopy

## Abstract

The comprehensive characterisation of complex, irreplaceable cultural heritage artefacts presents significant challenges for traditional analytical methods, which can fall short in providing multi‐scale, non‐invasive analysis. Correlative Multimodal Microscopy (CoMic), an approach that integrates data from multiple techniques, offers a powerful solution by bridging structural, chemical, and topographical information across different length scales. This paper provides a comprehensive review of the evolution, current applications, and future trajectory of CoMic within the field of heritage science. We present a historical overview of microscopy in heritage studies and detail the principles and advances of key techniques, such as electron, X‐ray, optical, and probe microscopies. This review presents practical applications through case studies on materials that include wood, pigments, ceramics, metals, and textiles. To aid CoMic uptake, we also provide user‐centric guides for researchers with diverse expertise. This review also examines the challenges that currently limit the widespread adoption of CoMic, challenges that include sample preparation, data correlation accuracy, high instrumental and resource costs, and the need for specialised interdisciplinary expertise. Although CoMic is a transformative methodology for artefact analysis and conservation, its full potential will be realised through future developments in accessible instrumentation, standardised protocols, and the integration of AI‐driven data analysis. This review serves as a critical resource and roadmap for researchers, conservators, and institutions looking to harness the power of correlative microscopy to preserve our shared cultural legacy.

## 1 INTRODUCTION

In the intricate tapestry of cultural heritage studies, the role of microscopy has evolved from a mere observer's tool to a pivotal instrument, enabling not just the viewing but also the understanding of the secrets locked within historical artefacts. This evolution is particularly evident in the field's response to specific challenges, such as the detailed analysis of complex material compositions and the non‐invasive examination of delicate artefacts, where traditional methods fall short.

This review paper delves into the realm of Correlative Multimodal Microscopy (CoMic), a technique heavily utilised for biological research, yet to be fully harnessed in the study of cultural heritage. Its relevance for heritage studies cannot be understated. Heritage artefacts are repositories of historical, artistic, and cultural narratives. These artefacts, ranging from ancient manuscripts to sculptures and paintings, are often complex composites of diverse materials.^1^, ^2^ Understanding their composition, structure, and degradation mechanisms is crucial for conservation and restoration efforts.^3^ CoMic emerges as a powerful solution to these challenges by offering a comprehensive and multi‐layered analysis that conventional single‐method approaches cannot provide^4^, ^5^ The term ‘correlative’ refers to combining multiple microscopy techniques to study the same specimen or region of interest within a specimen, to yield different information. Since different microscopy techniques each have their own advantages and limitations, such as spatial resolution and field of view, the resulting data often spans multiple length scales.^6^ ‘Multimodal’ refers to the applied combination of imaging, spectroscopy, and diffraction techniques to a specimen to yield additional information, which could involve electrons, X‐rays, optical photons, and neutrons.^7^ By harnessing the power of these various microscopic techniques, CoMic leverages their strengths to offer an unprecedented ability to examine artefacts at multiple scales and dimensions, revealing insights that were once unattainable.^8^ This technique transcends the limitations of traditional single microscopy, which provides a limited perspective confined to specific scales or dimensions.^9^

The objective of this review is to provide a comprehensive overview of the advances in CoMic and its application in the field of cultural heritage. By examining the methodological advancements, discussing significant case studies, and exploring the challenges and future perspectives, this paper aims to highlight the transformative impact of this technology in understanding and preserving our cultural legacy. Tracing the trajectory of this field, this review begins with a historical overview, charting the evolution of microscopy in heritage studies. From the early days of optical microscopy (OM) to the advent of advanced techniques like Scanning Electron Microscope (SEM) and Transmission Electron Microscope (TEM), the journey reflects a growing sophistication in imaging capabilities and analysis techniques.^10^ This historical context sets the stage for understanding the paradigm shift brought about by the introduction of CoMic approaches.

The methodological advances section delves into the technical aspects of various microscopy techniques. Each technique is explored in terms of its principle, advancements, and specific role in the correlative framework. The focus is on how these diverse techniques complement each other, allowing researchers to glean multi‐dimensional information from the same region of an artefact.^11^, ^12^ Case studies and applications form the core of the review, showcasing real‐world examples where CoMic has provided groundbreaking insights. These case studies not only illustrate the practical applications of these techniques but also underscore their significance in solving complex puzzles in cultural heritage conservation.

Despite its remarkable capabilities, CoMic is not without challenges. Technical limitations, methodological complexities, access to equipment, and the need for specialised expertise are some of the hurdles faced in this field. These challenges are addressed, providing a balanced perspective of the current state of the art, before looking towards the future to explore emerging trends and potential advancements in the field. The continual development of new imaging techniques and analytical tools promises to further enhance the capabilities of CoMic, opening new horizons in cultural heritage studies. By providing a detailed analysis of its development, applications, and future potential, this paper seeks to contribute to the ongoing discourse in this fascinating intersection of science, art, and history.

**FIGURE 1:**
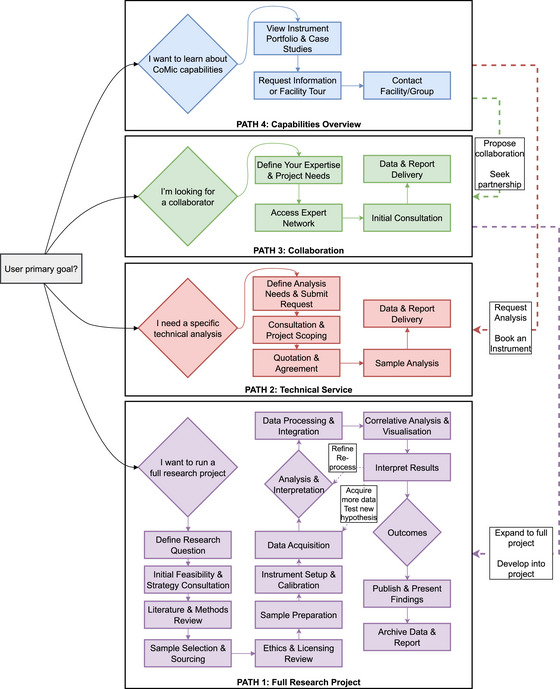
A user‐centric decision‐making flowchart for CoMic engagement. The diagram details four distinct models: Path 1 (purple) for comprehensive research projects, Path 2 (red) for specific technical analyses, Path 3 (green) for initiating collaborations, and Path 4 (blue) for new users. Feedback loops show the iterative nature of research, while connecting arrows illustrate potential user journeys between paths.

To aid researchers from diverse backgrounds in navigating the complexities of CoMic, two complementary guides are provided. Table 1 outlines potential user profiles from a heritage perspective, while Figure 1 presents a corresponding flowchart of engagement pathways. This schematic illustrates how individuals with varying expertise can approach a CoMIC study, highlighting key decision points and resource needs. While not exhaustive, these tools serve as a practical guide for initiating and executing CoMic investigations.

**TABLE 1:** User profiles and possible solutions.

User type	Problem area	Issue description	Possible solution
Heritage expert, no microscopy knowledge	Hardware selection, software understanding	Identifying suitable techniques or software for data integration.	Provide a cross‐reference guide linking heritage materials to techniques and software examples.
Balanced generalist	Time/IP/resource underestimation	May overlook licensing issues or underestimate time needed for data correlation.	Include IP alert prompts and estimated resource/time charts.
Microscopy expert, no heritage	Ethics, application relevance	Unaware of ethical considerations or practical relevance to heritage.	Integrate heritage‐specific ethics checklist and application framing prompts.
Conservator	Method selection	Unfamiliar with emerging, non‐destructive imaging options.	Provide a decision tree mapping object fragility and material to suitable methods.
Curator or collections manager	Experimental design	Not involved in method planning; concerned with object risk.	Insert object risk review prompt before experimental design.
PhD student/ECR	Literature search, data archiving	Unfamiliar with databases or archiving in heritage science.	Provide search templates and repository recommendations.
Instrument specialist/technician	Application alignment	May generate data without checking application relevance.	Prompt coordination with heritage stakeholder or PI.
Data scientist/imaging analyst	Acquisition details, software compatibility	Unclear on data formats and acquisition metadata needs.	Include acquisition metadata guidance and format compatibility matrix.
Funding applicant	Feasibility, IP planning	May not plan for IP, licensing, or hardware feasibility.	Embed early IP alert and cost/feasibility checklist.
Museum educator/outreach lead	Purpose framing	Unfamiliar with framing analytical questions for CoMic.	Include example use cases for outreach and education.
Commercial partner	Integration expectations	Unaware of interoperability or correlation requirements.	Include integration guidance and expected data standards.

## 2 HISTORICAL OVERVIEW

The integration of microscopy into cultural heritage studies has reflected a progressive evolution in both technology and methodology, mirroring the increasing complexity of artefact analysis and preservation. Microscopy's journey in this field began with the use of simple optical microscopes, where early investigators employed these devices to unveil previously invisible material characteristics, enhancing the understanding of artefacts' microstructures and providing initial insights into their composition. The invention of the compound microscope in the 17th century by Antonie van Leeuwenhoek marked a pivotal moment, enabling the exploration of fine details, such as weave patterns in textiles or brush stroke textures in paintings. This early work laid the foundation for more advanced studies that followed over the centuries. The 20th century witnessed a significant leap forward with the development of electron microscopy. The introduction of the TEM by Ernst Ruska and Max Knoll in the 1930s, followed by the SEM in the 1940s, revolutionised microscopy by providing far greater resolution than optical microscopes. These advancements enabled detailed imaging of artefact surfaces and internal structures at micro‐ and nano‐scales, leading SEM to become a mainstay in cultural heritage research by revealing crucial information about manufacturing techniques, wear patterns, and degradation processes.^10^, ^13^ As microscopy techniques advanced, the latter half of the 20th century saw the rise of analytical methods, including Energy Dispersive X‐ray Spectroscopy (EDS) integrated with SEM for elemental analysis.^14^ Non‐destructive chemical characterisation techniques, such as Fourier Transform Infrared (FTIR) Microscopy and Raman Microscopy, further enhanced the ability to study artefacts, allowing researchers to identify their chemical compositions without damaging the materials.^15^, ^16^

The turn of the millennium marked a rapid acceleration in imaging technology and computational analysis. Techniques such as Confocal Laser Scanning Microscopy (CLSM) and X‐ray Computed Tomography (CT) began offering non‐invasive three‐dimensional (3D) imaging, revealing internal structures without the need for physical sectioning. Innovations like the Focused Ion Beam (FIB) technique, often coupled with SEM, enabled precise cross‐sectioning and imaging at the nanoscale, facilitating in‐depth studies of material microstructure.^17^, ^18^ Recognising the limitations of single techniques, the concept of correlative microscopy gained momentum. This approach involves using multiple microscopy modalities to study the same artefact region, combining complementary data to form a comprehensive analysis. For example, SEM images can be paired with TEM, Raman spectroscopy, and X‐ray CT data to provide a multi‐scale, multidimensional view of an artefact's surface topography, material composition, and internal structure.^19^, ^20^

**FIGURE 2:**
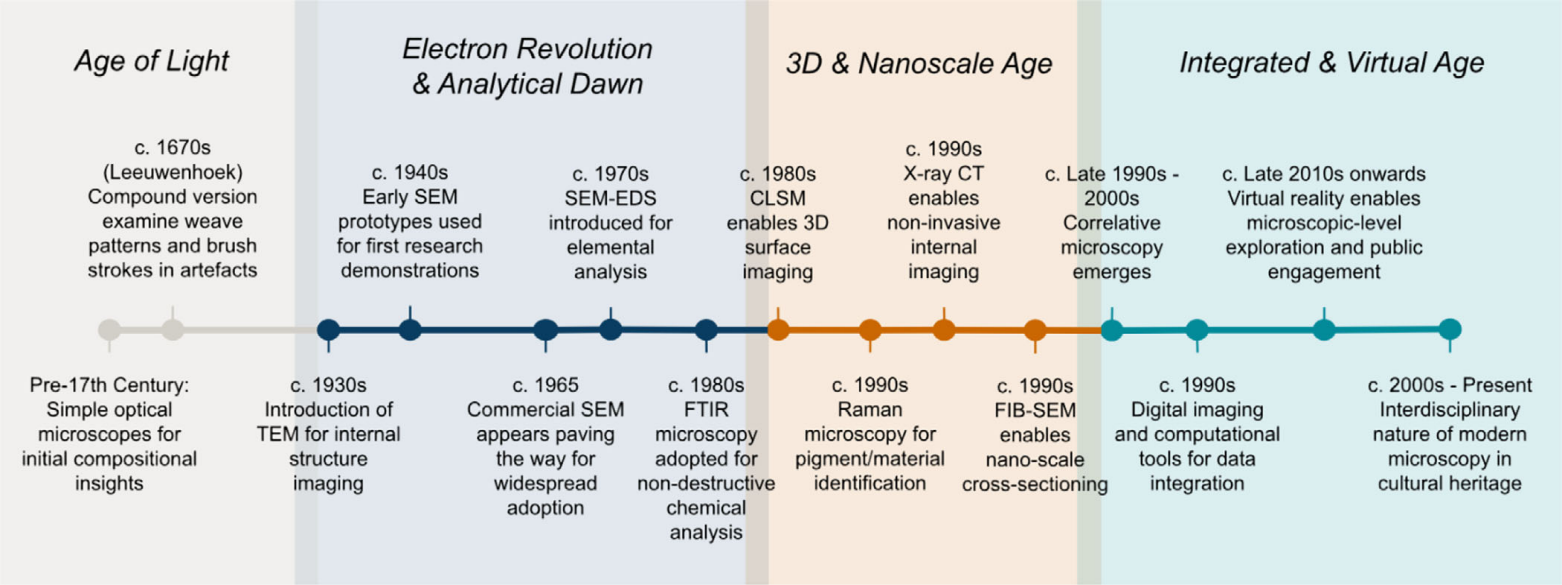
A timeline illustrating key developments in the field of correlative multimodal microscopy and associated imaging techniques.

The modern era of microscopy in cultural heritage is distinguished by its interdisciplinary nature, blending insights from materials science, chemistry, biology, and conservation. Advances in digital imaging and computational tools have facilitated the integration of diverse data sets, enabling the sophisticated visualisation and analysis of correlative microscopy data. Emerging technologies, such as virtual reality, are starting to offer virtual tours of artefacts at the microscopic level, making detailed structures accessible to both researchers and the public. As the field continues to advance, there is an increasing emphasis on non‐destructive analysis and the growing accessibility of advanced microscopy facilities. This trajectory suggests an exciting future for cultural heritage studies, with ongoing innovations in imaging techniques and data processing poised to unlock new insights into the material culture of the past. The historical progression from simple optical magnifiers to complex, correlative multimodal systems highlights a continuous quest to deepen our understanding of the artefacts that define human history. This historical progression, from simple optical magnifiers to complex, correlative multimodal systems, is summarised in the timeline in Figure 2.

## 3 METHODOLOGICAL ADVANCES

The field of cultural heritage studies has significantly advanced, driven by the continuous evolution of microscopy techniques. These advancements have allowed for unprecedented visualisation and analysis of artefacts, enhancing our understanding of their historical context, composition, and conservation needs. Below, we explore key microscopy techniques in terms of their principles, recent innovations, and their role within the CoMic framework.

### 3.1 Electron microscopy

**FIGURE 3:**
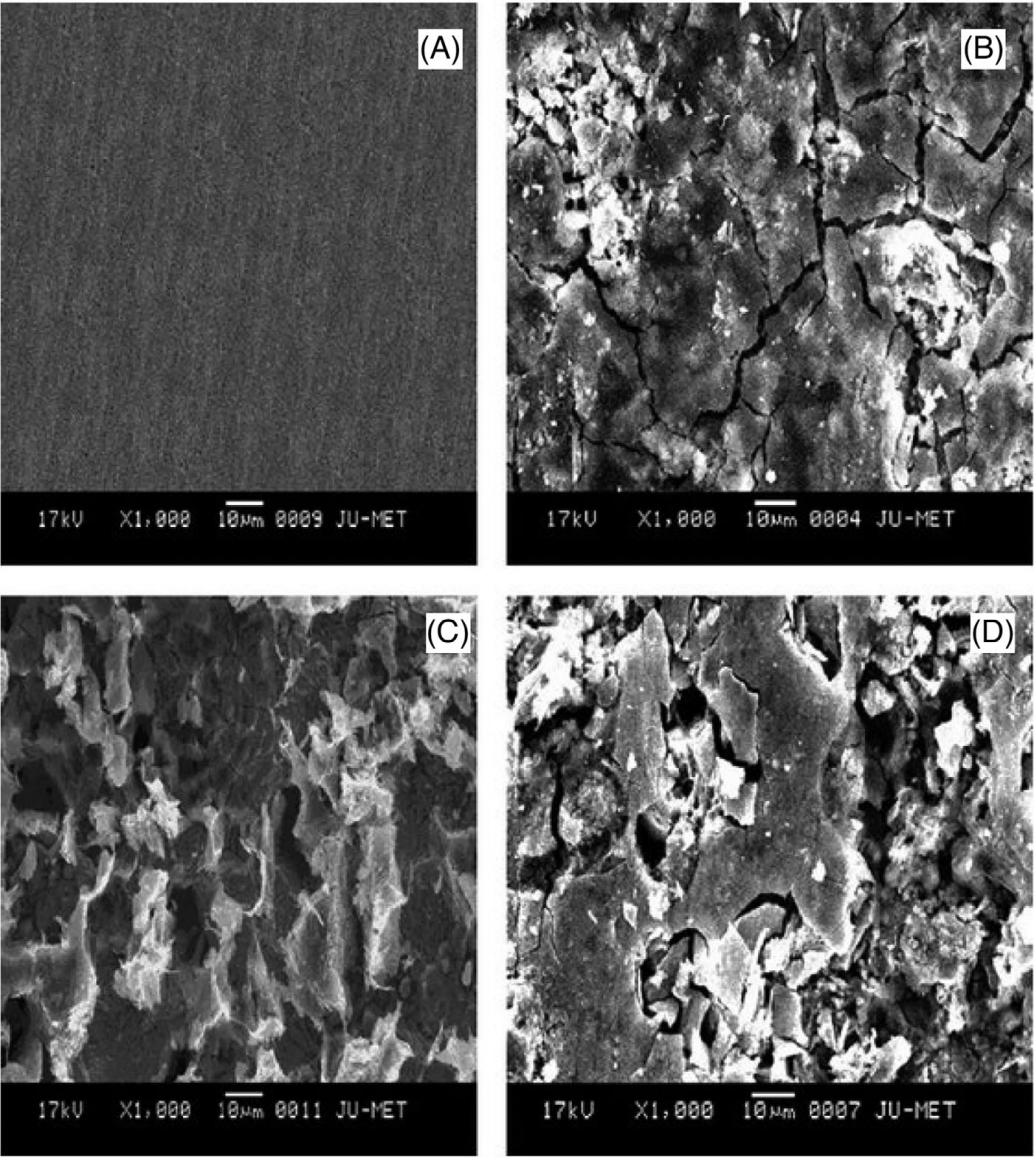
SEM images of corroded steel: (A) before corrosion, (B) in ${\mathrm{Na}}_{2}{\mathrm{SO}}_{4}$, (C) in ${\mathrm{Na}}_{2}{\mathrm{SO}}_{4}$ and ${\mathrm{Na}}_{2}S$, and (D) in ${\mathrm{Na}}_{2}{\mathrm{SO}}_{4}$, ${\mathrm{Na}}_{2}S$ and NaCl. Reproduced from Ref. 32 under CC BY 4.0.

Electron microscopy (EM), including SEM and TEM, plays a crucial role in cultural heritage studies.^19^ These techniques provide high‐resolution insights into both surface and internal structures, which are invaluable for understanding the material composition, wear patterns, and degradation processes of artefacts. SEM is renowned for its detailed surface imaging capabilities. By using secondary and backscattered electron detectors, SEM can generate high‐resolution images of artefact surfaces, revealing textures, material compositions, and structural changes over time,^20^, ^21^ an example of this can be seen in Figure 3. When combined with EDS, SEM also provides elemental analysis, essential for assessing artefact conservation status.^22^ TEM, with its superior spatial resolution, examines the internal microstructure at the atomic level, making it ideal for thin sections of materials, such as ancient ceramics or metal fragments.^23^ Recent developments in volume EM, such as Serial Block‐Face SEM (SBF‐SEM) and Focused Ion Beam SEM (FIB‐SEM), allow for 3D reconstructions of artefacts. These techniques are transformative for visualising the internal microstructures of porous ceramics, layered metal objects, and other complex materials, providing an in‐depth understanding of artefact fabrication and deterioration.^24^, ^25^ Advancements like Cryo‐Electron Microscopy (Cryo‐EM) have enabled the study of artefacts in a near‐native state. Cryo‐EM minimises sample damage and provides critical insights into fragile organic materials and hydrated archaeological finds, which conventional methods may alter.^26^, ^27^ Multi‐beam SEM technology, using multiple electron beams, accelerates data acquisition, making it possible to image large artefact surfaces at high resolution more efficiently. This is particularly useful for the examination of larger archaeological fragments or intricate details in decorative objects.^28^ In the correlative framework, EM provides detailed microstructural and compositional data that can be integrated with optical, X‐ray, and other microscopy techniques. This multi‐modal approach enables a comprehensive understanding of both the surface and internal characteristics of artefacts, offering essential insights into their preservation and historical significance.^29^, ^30^, ^31^

Figure 3 shows the increasing damage to steel produced by increasingly aggressive corrosive materials. Before the corrosion the surface of the steel appears to be smooth, with limited irregularities. If an EDS scan was to be performed on this surface we would expect to see peaks related to Fe and C, as well as any alloying elements depending on the type of steel used. Once the steel has been exposed to a corrosive agent, the first of which is ${\mathrm{Na}}_{2}{\mathrm{SO}}_{4}$, the surface of the metal is visibly damaged in the SEM image. The metal now has an uneven surface and cracks. An EDS scan performed on this surface would highlight the presence of S and O due to the presents of sulphate based corrosion products and oxidation. The addition of ${\mathrm{Na}}_{2}S$ to the surface of the steel would introduce sulphide ions. At this point the SEM image shows a heavily corroded surface. EDS would show an increased content of S. The final product added to the steel is NaCl, which Figure 3D shows to to have aggressively destroyed the surface of the steel. EDS at this stage would detect Cl, S and O, indicating complex corrosion.

### 3.2 X‐ray microscopy

**FIGURE 4:**
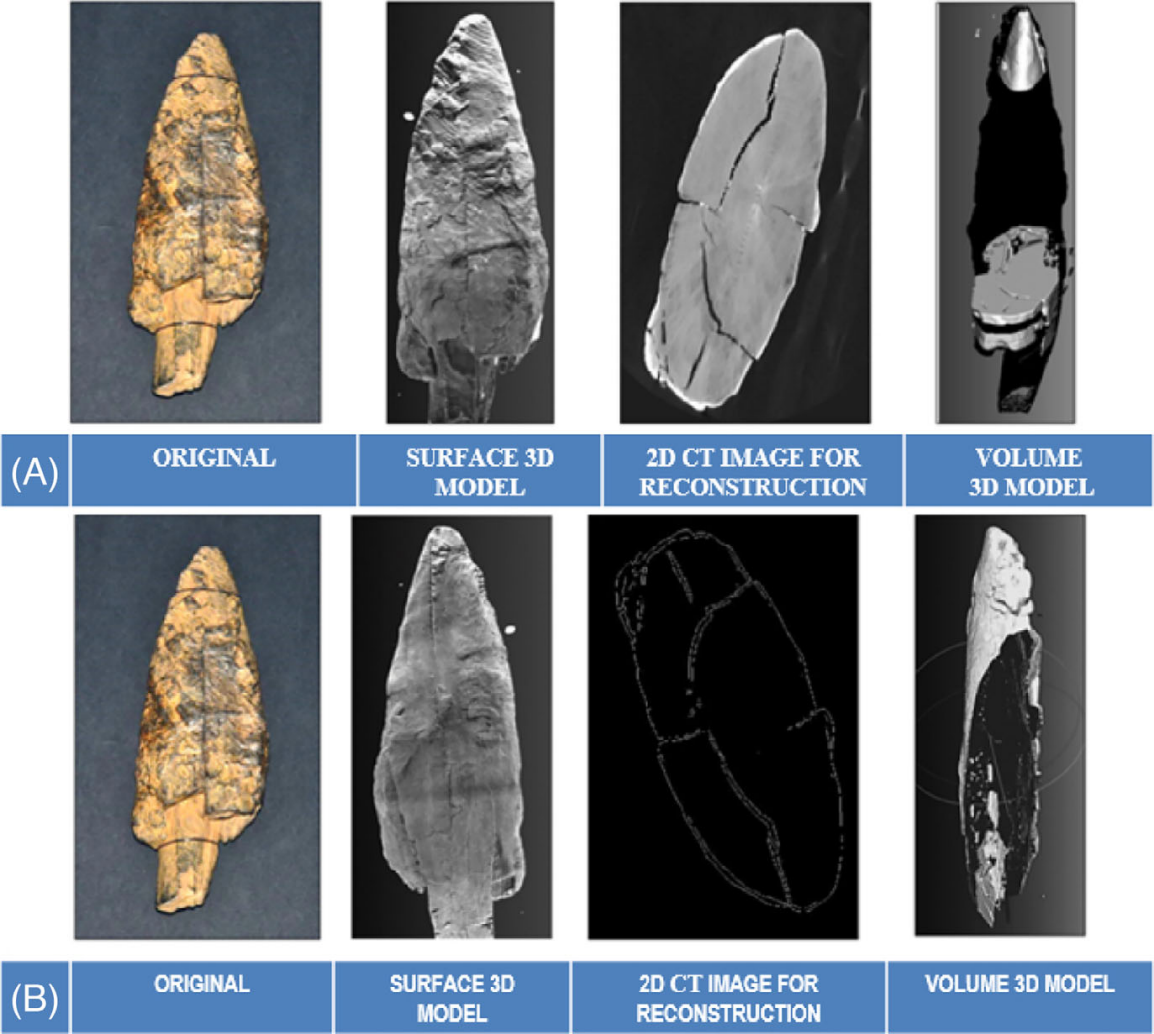
Reconstruction techniques applied to a 40,000‐year‐old Palaeolithic wooden point. Results shown: (A) direct reconstruction using the dAR3D algorithm, and (B) segmentation‐based reconstruction using the sAR3D algorithm. Reproduced from Ref. 42, licensed under CC BY 4.0.

X‐ray CT has emerged as a key technique for cultural heritage studies due to its ability to produce non‐invasive, 3D reconstructions of artefacts.^33^ By combining multiple X‐ray images captured during object rotation, CT generates cross‐sectional images that reveal the internal structure without damaging the artefact. Initially developed for medical applications, X‐ray CT has since been adapted for cultural heritage, particularly for wooden objects, metals, and ceramics.^34^, ^35^, ^36^, ^37^ However, dense metal artefacts present challenges, such as occlusion and streaking artefacts, which complicate image reconstruction.^38^ Recent advancements, such as post‐processing methods and improved scanning hardware, have addressed some of these limitations, enabling clearer imaging of complex materials like terracotta and glass,^39^ Figure 4 shows the use of this technology on a wooden point. CT scanners vary in resolution, from millimetre‐scale (used in clinical scanners) to micron and sub‐micron resolution (lab‐based and synchrotron facilities). This range allows researchers to choose the appropriate level of detail based on the artefact's material and preservation needs. X‐ray CT is particularly useful for studying delicate wooden objects, where techniques like dendrochronology and wood identification are essential for conservation and dating.^40^, ^41^

### 3.3 Optical microscopy

**FIGURE 5:**
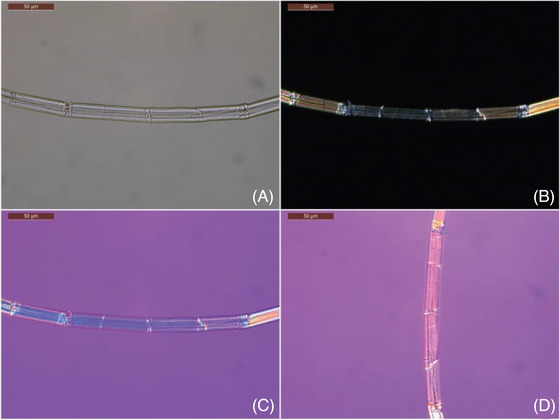
Transmitted light (A) and polarised light microscopy (B–D) images of a Viking Age textile fibre from burial B4864g,h. White light reveals surface morphology (A), while crossed polars (B) highlight extinction behaviour. Use of red‐plate compensator under PLM confirms fibre birefringence and dye response in 0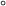 (C, Indigo II) and 90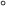 (D, Orange I) positions. Adapted from Ref. 54, under CC BY 4.0

There are a number of optical techniques that have been widely used to analyse heritage materials. Confocal microscopy provides high‐resolution, three‐dimensional imaging of surface layers and internal structures without the need for physical sampling,^43^, ^44^, ^45^ while fluorescence microscopy has been used to detect organic compounds and degradation products in artefacts, offering molecular‐level insights with minimal sample disruption. Polarised light microscopy (PLM), as seen in Figure 5 proves invaluable for studying crystalline materials, revealing key structural details in pigments, minerals, and varnishes. For broader surveys, widefield microscopy remains effective in imaging large areas with high contrast. Super‐Resolution Microscopy techniques, such as stimulated emission depletion and structured illumination, extend beyond the diffraction limit, providing unprecedented detail at the nanoscale, while dark field enhances contrast for specimens where traditional imaging may fail, revealing subtle structural features. While not microscopy, techniques that utilise optical photons for spectroscopy, such as Raman spectroscopy, are also crucial. Raman is particularly valuable for identifying pigments, minerals, and organic compounds in artefacts, such as paints and ceramics.^46^, ^47^, ^48^ The non‐invasive nature of Raman imaging makes it an appealing choice for analysing precious historical objects, but users should be aware of the risks involved: focused laser beams used to gather data from the specimen can cause localised heating or photochemical degradation if the power and wavelength are not appropriately chosen. To mitigate these risks low‐powered lasers are used alongside raster scanning techniques to minimise surface exposure.^49^, ^50^ The potential for surface damage depends on several factors, including the laser wavelength and power, as well as the optical and thermal properties of the sample. Materials with low thermal conductivity or high absorption at specific wavelengths are particularly susceptible to damage. Careful selection of laser parameters and, where possible, preliminary testing on inconspicuous areas or reference materials are recommended to ensure safe analysis conditions. Fourier Transform Infrared (FTIR) spectroscopy reveals the chemical composition of organic materials like varnishes, binders, and degradation products. This is critical for understanding an artefact's condition and informing conservation strategies.^51^, ^52^, ^53^ When integrated into a correlative framework, these techniques enhance the depth and accuracy of analysis, offering a multi‐dimensional view of artefact composition and condition, providing molecular, chemical, and structural information in a complementary manner. Figure 5 shows images of a Viking Age textile fibre under transmitted and polarised light microscopy. This figure highlights how PLM can reveal critical structural and compositional information from heritage textiles. The transmitted light image in Figure 5A provides insight into the fibre's surface morphology, while the crossed polars in Figure 5B reveal extinction behaviour resulting from crystalline alignment within the fibre. Figure 5C and D, taken using a red‐plate compensator, demonstrates birefringence and help distinguish between different dye compounds based on their optical response at 0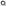 and 90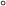 orientations.

### 3.4 Neutron microscopy

**FIGURE 6:**
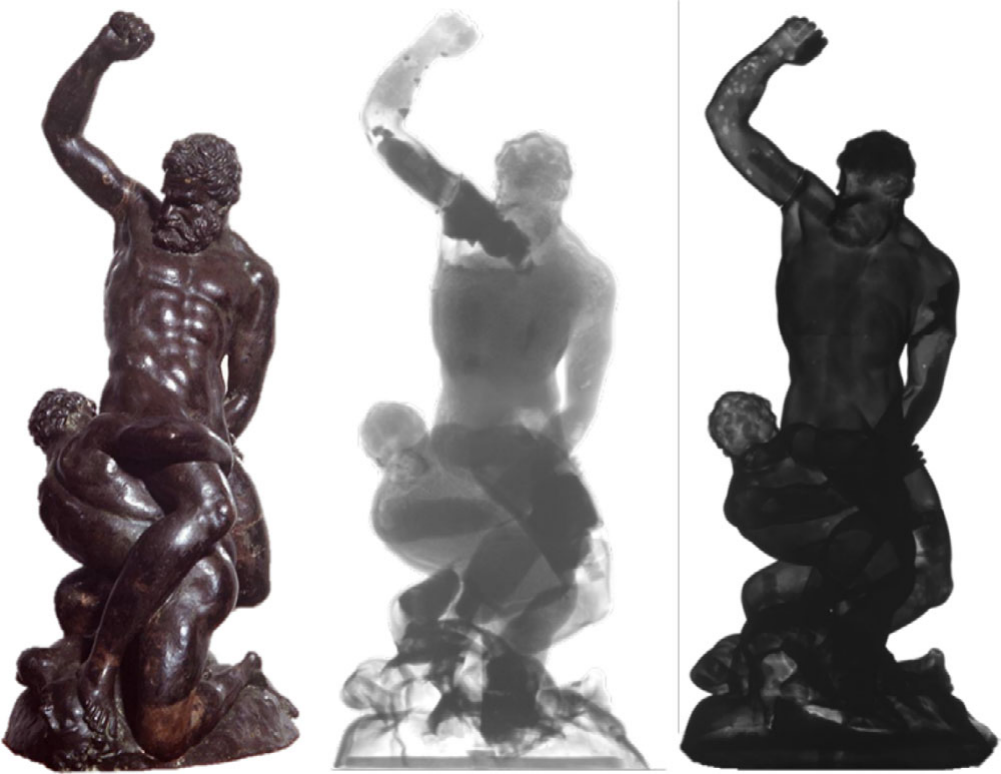
A multimodal view of the sculpture *Samson Slaying a Philistine*. Pictured are (left) a visual reference photograph, (centre) a neutron radiograph, and (right) an X‐ray radiograph. The neutron image reveals organic fillers and restoration materials, while the X‐ray image highlights dense metal, showing casting defects and internal supports. Together, they provide a comprehensive, non‐invasive understanding of the object's structure and history. Reproduced from Ref. 62 under CC BY 3.0

Neutron imaging techniques offer unique advantages in cultural heritage studies, especially for organic and composite materials. Neutron radiography and tomography can reveal internal structures in artefacts that are invisible to X‐ray techniques, such as the moisture content in wood or organic inclusions in ceramics.^55^ While less commonly used than other microscopy methods, neutron imaging provides complementary data in the correlative framework, particularly in cases where X‐rays fail to penetrate dense materials,^56^, ^57^ an example of these differences can be seen in Figure 6. Recent advancements in neutron imaging have increased its resolution and applicability, making it more effective for non‐invasive analysis of complex artefacts.^58^ Techniques developed at facilities such as the NEUTRA and XTRA at PSI have enabled improved visualisation of mineral and organic inclusions, enhancing artefact characterisation.^59^ Neutron imaging's sensitivity to hydrogen makes it particularly effective for studying organic materials like wood and textile fibres, where hydration levels and internal compositions are critical for conservation.^60^ Neutron tomography's 3D imaging capabilities allow for a detailed examination of composite artefacts, providing invaluable insights into layered or soil‐encased objects that are otherwise difficult to study non‐invasively.^61^

### 3.5 Atomic force microscopy

**FIGURE 7:**
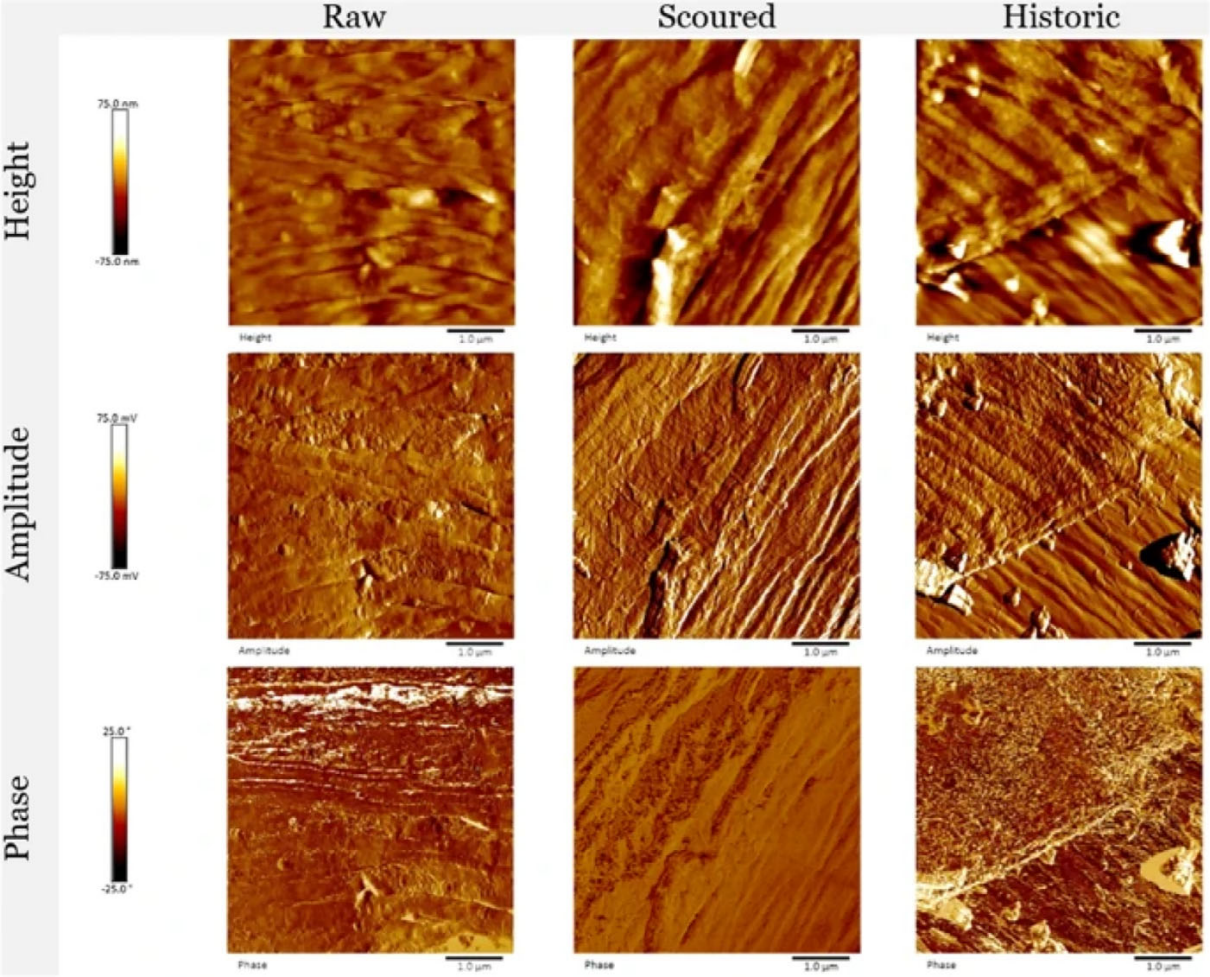
AFM height images of cotton fibres subjected to different bleaching treatments, showing changes in fibre morphology and surface topography at the nanoscale. Reproduced from Ref. 68 under the terms of the Creative Commons Attribution 4.0 International Licence (CC BY 4.0).

Atomic Force Microscopy (AFM) is a valuable tool in cultural heritage studies, particularly for analysing surface properties at the nanoscale. AFM measures topography, texture, and mechanical properties, which are crucial for assessing the state of preservation and degradation in materials such as metals, ceramics, and historical paintings,^63^ these abilities can be seen in Figure 7. AFM operates under ambient conditions without extensive sample preparation, making it suitable for delicate artefacts.^64^ By providing mechanical insights, such as surface roughness or stiffness, AFM complements other imaging techniques, offering a more comprehensive understanding of artefacts in the correlative framework.^65^ Techniques like AFM‐IR further extend AFM's capabilities by enabling nanoscale chemical mapping, adding valuable information about the material composition of heritage objects.^66^ Advanced AFM methods, such as 3D AFM, offer atomic and molecular‐resolution imaging, allowing for detailed topographic mapping and texture analysis of fragile surfaces. This ability to operate at the nanoscale is particularly advantageous for monitoring preservation needs and detecting early signs of degradation in cultural heritage objects.^67^ Figure 7 shows AFM's sensitivity, as it can detect nanoscale differences on the surface of cotton fibres as a result of different bleaching treatments. For example, the raw fibres exhibit irregular topography with limited fibrillar definition, scoured fibres show greater alignment and smoother surfaces, while historic fibres display disrupted, heterogeneous structures consistent with ageing. The three contrast modes further emphasise these differences: height images reveal topography, amplitude highlights edge sharpness and texture, and phase captures local variations in mechanical properties such as stiffness.

### 3.6 Other microscopy techniques

**FIGURE 8:**
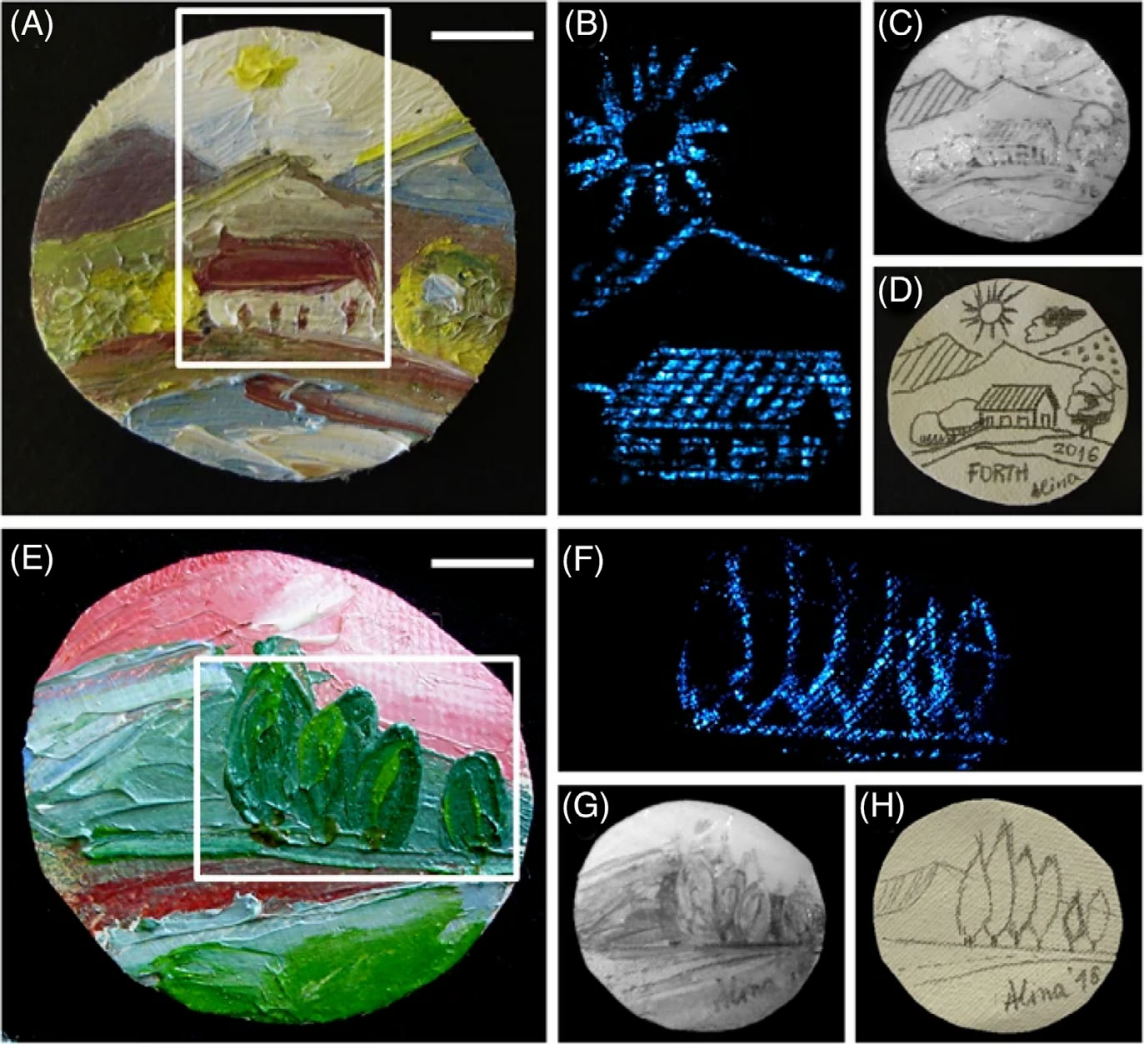
Photoacoustic Microscopy (PAM) demonstrating the non‐invasive detection of hidden, carbon‐based pencil drawings beneath layers of paint on two different samples. The analysis of the first sample shows the bright‐field image of a painting depicting a house (A), the corresponding PAM image which clearly reveals the hidden pencil underdrawing (B), and a Near‐Infrared (NIR) image showing minimal contrast(C). The original reference sketch is also shown (D). A similar analysis of a second painting depicting trees (E) again shows the PAM image revealing the hidden drawing (F), while the NIR image fails to detect it (G). The reference sketch for the second painting is included for comparison (H). Reproduced under CC BY 4.0 from Ref. 77.

Other advanced microscopy techniques, such as Acoustic Microscopy, Oblique Illumination Spectral Microscope, Photoacoustic Microscopy (PAM), and Scanning Probe Microscopy (SPM), add further dimensions to the CoMic approach. Acoustic Microscopy uses ultrasound waves to probe internal structures, making it valuable for fragile or composite artefacts.^69^, ^70^ PAM offers insights into optical absorption properties, revealing hidden layers in manuscripts or paintings,^71^, ^72^ as seen in Figure 8. SPM techniques, like Scanning Tunnelling Microscopy (STM), enable atomic‐level surface imaging, which is particularly useful for metal artefacts.^73^, ^74^ Emerging techniques like Digital Holographic Microscopy (DHM) and Magnetic Resonance Imaging (MRI) provide non‐invasive analysis of transparent or semi‐transparent artefacts and organic materials. DHM is particularly effective for analysing transparent layers, while MRI excels in examining the internal architecture of wood, textiles, and parchments, contributing to conservation strategies without physical intervention.^21^, ^75^, ^76^ Advanced photoacoustic systems and adaptive optics have enhanced depth imaging capabilities, making it possible to analyse complex artefact structures in greater detail.

### 3.7 Image‐based simulation

**FIGURE 9:**
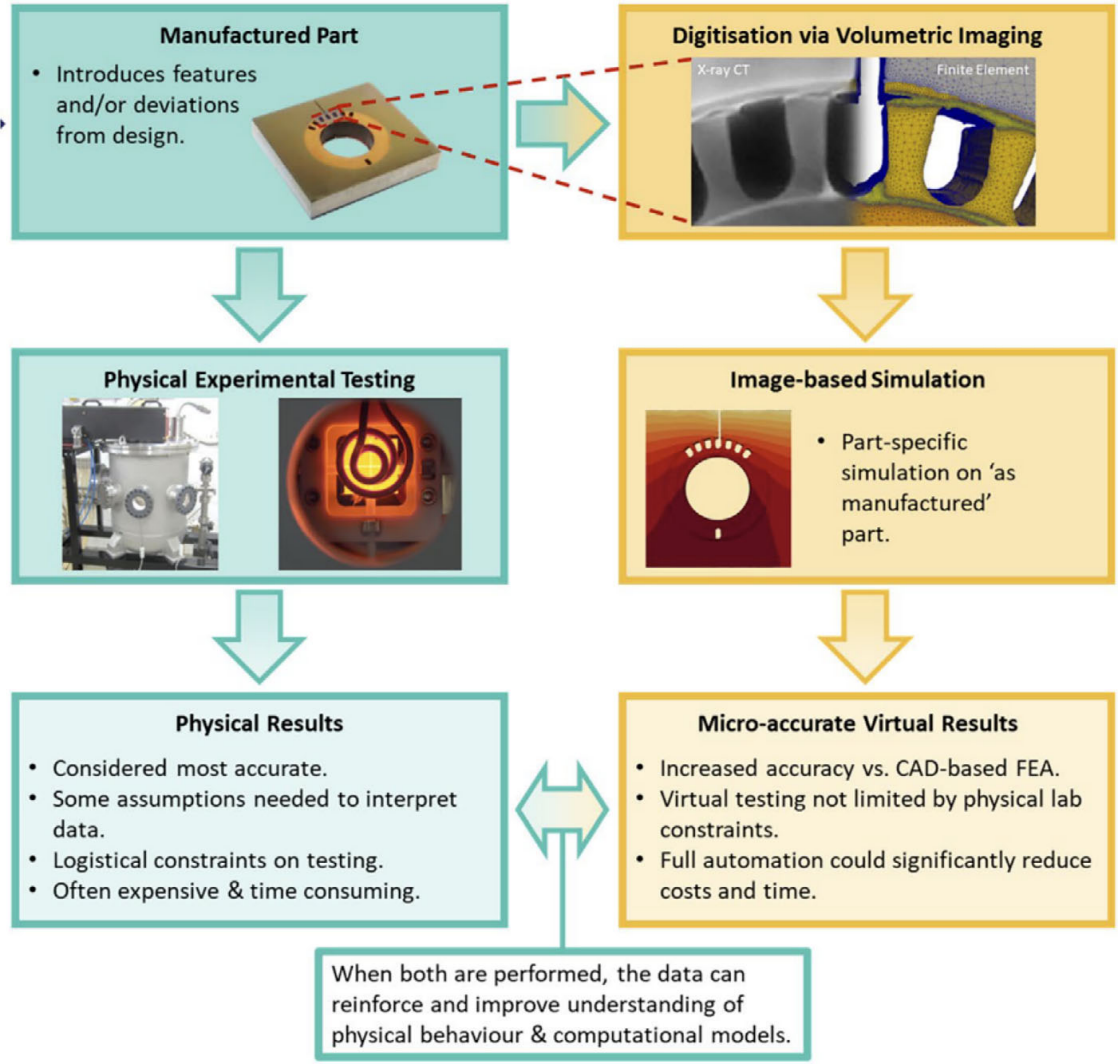
Flowchart illustrating the relationship between traditional ‘as‐designed’ simulation approaches and more recent ‘as‐manufactured’ virtual testing methods enabled by image‐based simulation. Adapted under CC BY 4.0 from Ref. 82.

Image‐based simulation, when integrated with microscopy data, enhances the interpretation of artefact behaviour over time. By simulating physical and chemical processes, researchers can visualise degradation mechanisms and assess artefact stability non‐invasively.^78^ This predictive capability allows for more targeted conservation strategies and informs decisions on long‐term preservation. Simulations based on X‐ray CT and other imaging techniques have proven particularly effective in predicting structural changes in fragile artefacts.^79^ High‐resolution 3D imaging and modelling techniques provide an essential basis for simulating structural conditions, with applications that include monitoring valuable artefacts like the Morgantina silver treasure and delicate ceramics.^80^, ^81^ While drawn from the high‐value manufacturing sector, the principles shown in the Figure 9 are directly transferable to heritage samples where morphological fidelity is critical.

### 3.8 Correlative multimodal microscopy

**FIGURE 10:**
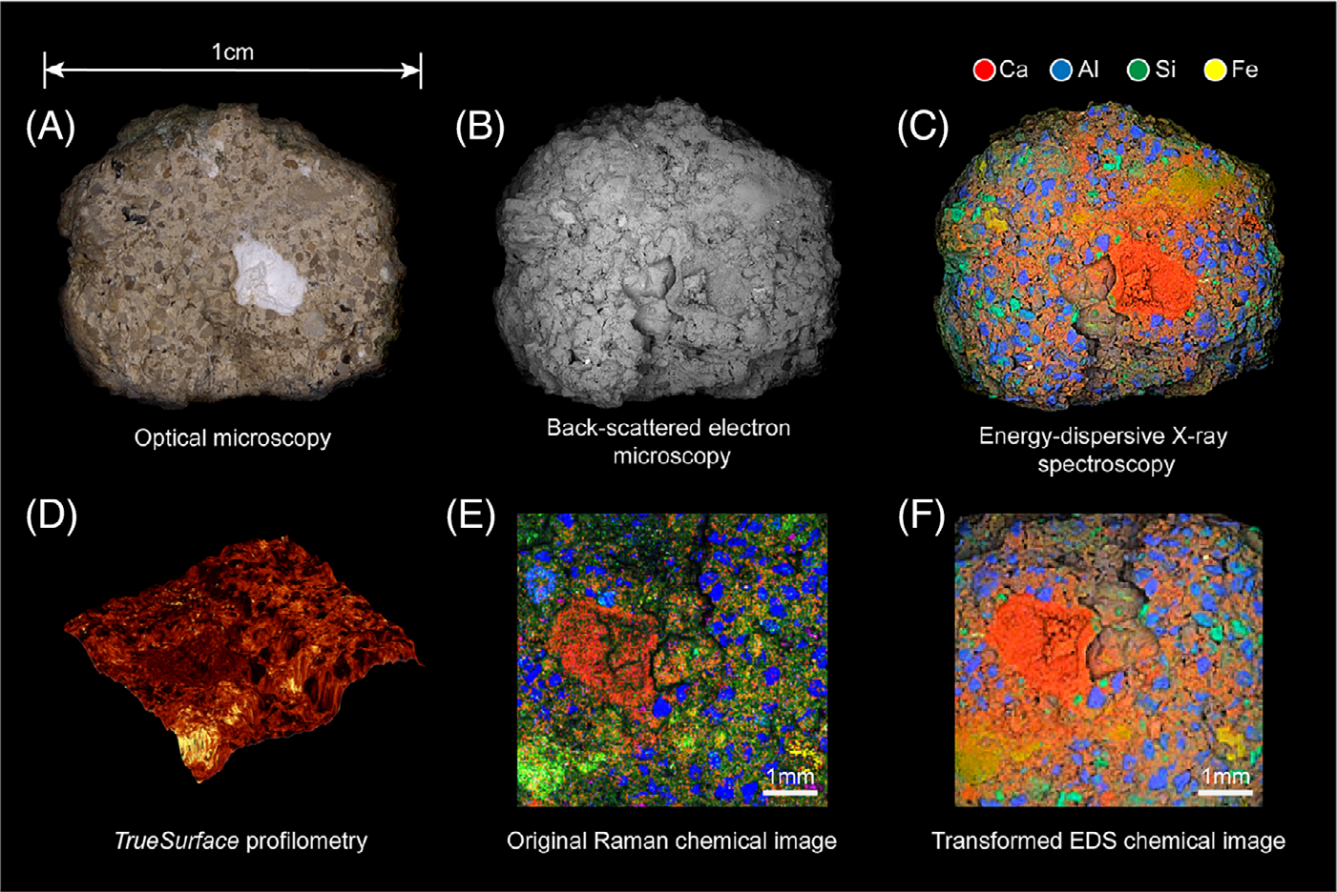
A wonderful example of CoMic applied to Roman Concrete. Reproduced from Ref. 89, originally published in PLOS ONE under a Creative Commons Attribution licence (CC BY 4.0).

The strength of correlative multimodal microscopy lies in its ability to combine diverse techniques, allowing for a comprehensive multi‐dimensional analysis of artefacts. By integrating data from electron, X‐ray, optical, neutron, and force microscopy, researchers can gain insights into both the surface and internal composition of artefacts, bridging the gap between structural, chemical, and mechanical properties,^65^, ^83^ an example of CoMic can be seen in Figure 10, highlighting the quantity of information achievable through CoMic. This approach is particularly valuable for cultural heritage applications, where synchrotron radiation and other accelerator‐based methods can offer exceptional analytical power. Synchrotron facilities now enable micro‐ and nanoscale characterisation of layered artworks and fossils via techniques such as X‐ray absorption spectroscopy (XAS), X‐ray fluorescence (XRF), and nano‐beam imaging,^84^ revealing compositional layering and degradation processes with unprecedented resolution.^85^ In parallel, ion beam techniques including proton‐induced X‐ray emission (PIXE) and nuclear microprobes provide non‐destructive in situ elemental analysis,^86^ exemplified by the AGLAE accelerator at the Louvre, which has been applied to trace element mapping in artworks.^86^, ^87^ Such methods substantially expand the analytical possibilities beyond conventional laboratory‐based X‐ray systems and provide essential insights into material stability and provenance. For instance, combining X‐ray microscopy with optical and chemical imaging techniques enables precise analysis of morphological and chemical changes in historical materials. By adopting a multimodal approach, conservationists can achieve a more holistic understanding of artefacts, supporting effective preservation practices and providing deeper insights into the historical significance of these objects.^88^ Figure 10 shows six different images of a piece of Roman Concrete. Between the six images the user can gather a sense of the morphology, elemental composition and distribution, and surface texture of the sample. Specifically, optical microscopy (a) gives a macroscopic view of the concrete surface, while back‐scattered electron microscopy (b) highlights density and compositional contrast. EDS mapping (c) reveals elemental distributions of Ca, Al, Si, and Fe, showing the spatial organisation of key phases. TrueSurface profilometry (d) captures the 3D topography of the rough concrete surface, while Raman chemical imaging (e) identifies mineral phases in situ. Finally, the transformed EDS chemical image (f) provides a direct correlation between Raman and EDS data, enabling validation and cross‐comparison of chemical and structural information.

## 4 COMIC APPLICATIONS BY MATERIAL

The real‐world impact of CoMic is best demonstrated through its practical application in analysing and preserving cultural heritage artefacts. This section highlights key case studies that showcase the breadth and versatility of CoMic across different material types. These examples illustrate how advanced microscopy techniques, integrated into a correlative framework, reveal crucial details about the composition, manufacturing methods, and degradation of artefacts, while also advancing conservation strategies.

### 4.1 Wooden artefacts

#### 4.1.1 Material properties and challenges

Wood has been widely used across cultures for crafting functional and ceremonial artefacts, from furniture and architectural elements to sculptures and burial objects. However, wood's organic composition makes it particularly vulnerable to environmental and biological decay. Over time, factors such as humidity, temperature fluctuations, and biological threats (like insects and fungi) can lead to structural and surface degradation, manifesting as warping, cracking, and insect‐induced hollows.^90^, ^91^ Understanding the internal and external condition of wooden artefacts without invasive sampling is essential in heritage conservation, as these artefacts often hold immense cultural and historical value.

#### 4.1.2 Potential CoMic techniques

**FIGURE 11:**
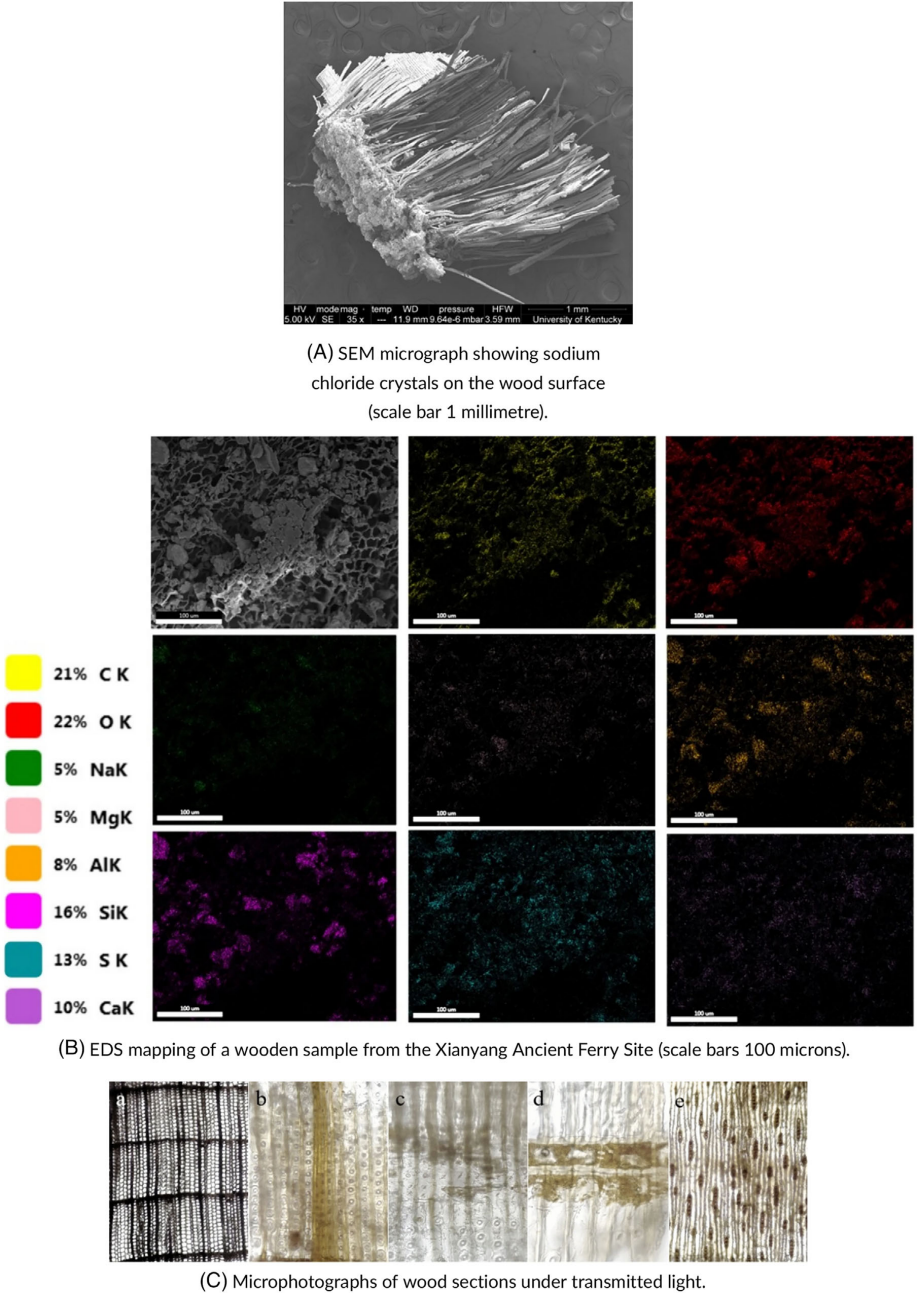
Microscopy images of wooden artefacts using SEM, EDS, and transmitted light techniques. Reproduced from Refs. 98, 99, 100, licensed under CC BY 4.0.

CoMic provides a robust suite of techniques for analysing the complex structure of wood. X‐ray CT is especially valuable in visualising internal features, such as voids, cracks, and other damage within the wood structure, without the need for physical disassembly.^92^ SEM can then be used to observe surface textures at a high resolution, capturing details like the wood fibres' alignment, insect damage patterns, and surface erosion caused by environmental exposure.^93^ Additionally, AFM can assess the surface roughness and elasticity of the wood, giving insight into its current mechanical properties.^94^ By integrating these methods, CoMic enables a thorough examination of both the surface and subsurface characteristics of wooden artefacts, presenting a non‐invasive yet comprehensive diagnostic framework. Figure 11 provides microscopic views of wooden artefacts, showing sodium chloride crystals on a surface via SEM (a), elemental mapping with EDS (b), and internal structures through transmitted light (c). These observations are conservation‐relevant: salt crystallisation identified by SEM (a) is a common driver of surface flaking, EDS mapping (b) clarifies the chemical nature of such deposits and contaminants, and transmitted light microscopy (c) reveals cellular structures that indicate fungal attack or previous treatment.

#### 4.1.3 Conservation implications

CoMic techniques offer a pathway to more precise, minimally invasive conservation strategies for wooden artefacts. For instance, X‐ray CT can pinpoint internal weaknesses and cracks that need immediate stabilisation,^95^ while SEM helps conservators assess the extent of surface damage and plan for preventive measures against further biological deterioration.^96^ AFM's mechanical insights enable conservators to make informed choices about consolidation agents, ensuring that any application reinforces the wood's strength without compromising its texture or appearance.^97^ By combining these techniques, CoMic allows conservators to map out degradation, stabilise fragile structures, and guide long‐term preservation, all while maintaining the artefact's historical and aesthetic integrity.

### 4.2 Pigments and paintings

#### 4.2.1 Material properties and challenges

Pigments and paintings, central to cultural heritage, often suffer from fading, discolouration, delamination, and sensitivity to environmental factors like humidity and light.^101^ The layered nature of painted surfaces and the delicate nature of historical pigments create challenges in diagnosing and conserving these artefacts without invasive sampling. For instance, fading or cracking may be indicative of pigment instability or past restoration attempts, requiring precise material identification to guide authentic preservation.^102^

#### 4.2.2 Potential CoMic techniques

**FIGURE 12:**
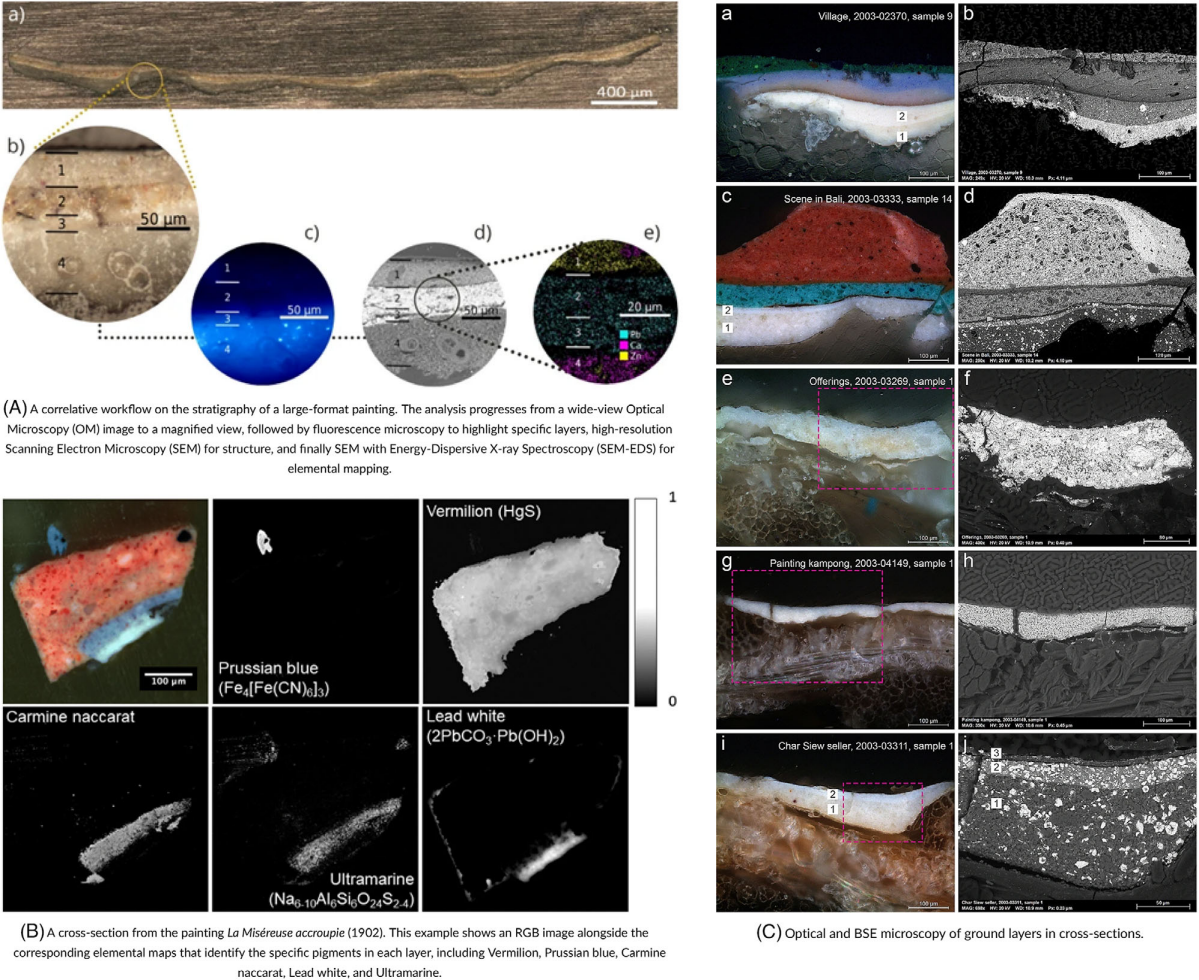
Examples of multimodal microscopy used for the analysis of paint cross‐sections. Reproduced from Refs. 108, 109, 110, licensed under CC BY 4.0.

CoMic techniques offer a multifaceted approach for analysing pigments and layered paint compositions. Raman microscopy is particularly valuable for identifying pigments based on their molecular spectra, offering insights into original materials and any restorations added over time.^103^ SEM provides high‐resolution imaging of paint layer morphology, revealing cracks, pigment detachment, and surface erosion.^104^ This can be complemented by FTIR spectroscopy, which detects organic compounds to analyse binder materials.^45^ Additionally, hyperspectral imaging offers a visual map of pigment distribution, identifying areas of degradation or discolouration.^105^ Together, these techniques provide a non‐invasive, comprehensive approach to characterising paint layers and identifying restoration requirements. The application of these techniques is demonstrated in Figure 12A and B, which shows both the stratigraphy of a paint cross‐section and elemental pigment mapping. In Figure 12B, the RGB image of the cross‐section is paired with elemental distribution maps that identify the pigments present in each layer. Vermilion HgS is mapped in the upper red region, Prussian blue ${\mathrm{Fe}}_{4}{\left[\mathrm{Fe}{\left(\mathrm{CN}\right)}_{6}\right]}_{3}$ in blue layers, carmine naccarat in pink regions, lead white $2{\mathrm{PbCO}}_{3}\cdot \mathrm{Pb}{\left(\mathrm{OH}\right)}_{2}$ in the light ground layers, and ultramarine ${\mathrm{Na}}_{8-10}{\mathrm{Al}}_{6}{\mathrm{Si}}_{6}O_{24}S_{2-4}$ in blue layers. Together, these maps show how multiple pigments were applied in distinct stratigraphic layers, providing both chemical identification and spatial context within the painting. These techniques allow for comprehensive analysis of material composition, structure and layering of historical paintings.

#### 4.2.3 Conservation implications

By combining CoMic techniques, conservators can accurately assess the authenticity of pigments, detect underlying degradation, and differentiate original materials from previous restorations. This informs treatments that aim to stabilise pigments, mitigate fading, and prevent further delamination. For example, Raman microscopy can determine the exact pigment composition,^106^ guiding precise colour‐matching in restorative applications. SEM's detailed imaging allows conservators to assess the stability of paint layers,^107^ enabling decisions that protect these artworks' visual and historical integrity. CoMic thus enables a targeted approach to restoration that respects historical authenticity while preserving material longevity. Further examples of optical and BSE microscopy being used to examine the ground layers in paint cross‐sections are presented in Figure 12C. This figure presents a combined optical and BSE microscopy image of a paint cross‐section enabling simultaneous visualisation of the layer structure and composition.

### 4.3 Glass and ceramics

#### 4.3.1 Material properties and challenges

Ceramic artefacts, often integral to archaeological and historical collections, present unique conservation challenges. Over time, they may develop microcracks, glaze deterioration, and mineral leaching due to exposure to environmental factors. Ceramics are also highly varied in composition – encompassing materials like clay, glazes, and sometimes pigments – each of which may respond differently to ageing and environmental stressors.^111^ Understanding these internal and surface‐level changes is crucial to inform preservation and restoration methods.

#### 4.3.2 Potential CoMic techniques

**FIGURE 13:**
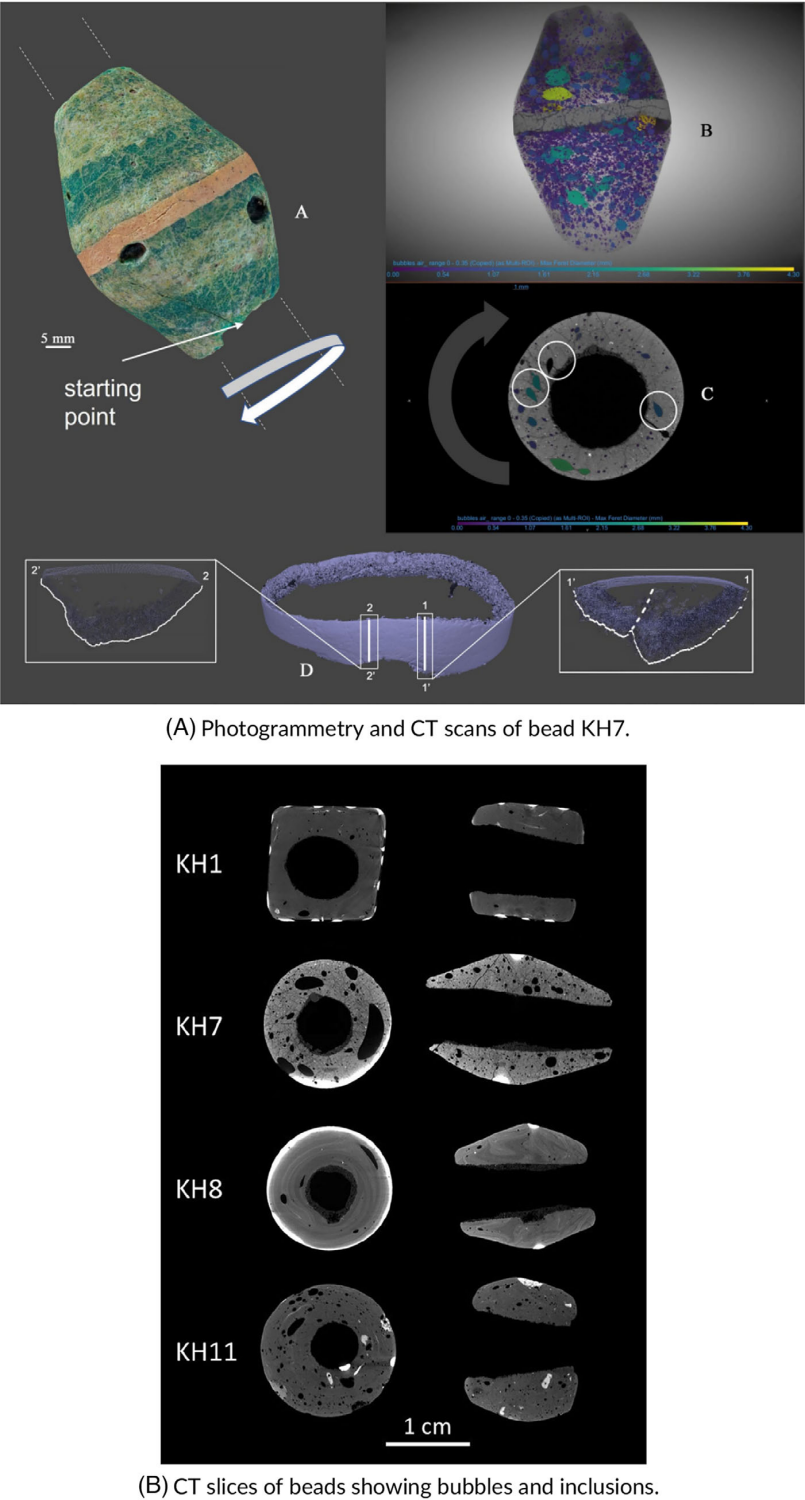
Microscopy and imaging of glass and ceramic artefacts using SEM, OM, and CT techniques. Reproduced from Ref. 117, licensed under CC BY 4.0.

X‐ray CT is particularly effective for identifying internal features of ceramics, such as microcracks and voids that are not visible on the surface.^112^ SEM allows for detailed surface examination, offering insights into the microstructure of glazes and clay materials, which are often sensitive to both physical stress and chemical degradation.^113^ EDS, often combined with SEM, provides elemental composition analysis, identifying the materials used in glazes and potential sources of degradation.^114^ By integrating these techniques, CoMic allows a thorough, non‐destructive analysis of ceramic artefacts, revealing information critical for preventive conservation. Figure 13 illustrates this using ancient glass beads. Figure 13A shows the integration of photogrammetry with CT scanning for bead KH7, enabling both external surface documentation and internal volumetric analysis. The CT slices (Figure 13B) reveal manufacturing details such as bubbles, voids, and mineral inclusions within several beads (KH1, KH7, KH8, KH11). These features provide insight into the production techniques, including blowing and moulding, and highlight internal heterogeneities that may later serve as points of weakness or degradation. Together, these complementary imaging approaches allow both the external appearance and the concealed internal structure to be assessed in a single workflow.

#### 4.3.3 Conservation implications

The insights gained from CoMic techniques guide conservators in detecting structural weaknesses, understanding glaze composition, and recognising signs of environmental impact. For instance, EDS can detect chemical alterations in glazes that might indicate mineral leaching,^115^ which in turn can help inform stabilisation treatments. X‐ray CT aids in the assessment of internal voids and fractures, enabling conservators to decide on the need for structural reinforcement.^116^ As shown in Figure 13B, features such as bubbles, voids, and inclusions provide evidence of original manufacturing practices but also indicate internal heterogeneities that may compromise long‐term stability. Recognising these concealed vulnerabilities allows conservators to anticipate failure points and apply targeted interventions. This integrated approach supports a conservation strategy that respects the ceramic's original structure and appearance while ensuring its long‐term preservation.

### 4.4 Metal artefacts

#### 4.4.1 Material properties and challenges

Metal artefacts, such as ancient tools, sculptures, and weapons, are highly vulnerable to corrosion and environmental degradation. Exposure to factors such as humidity, pollution, and fluctuating temperatures can accelerate oxidation and lead to structural weaknesses.^118^ CoMic offers a non‐invasive method to assess metal degradation, providing essential insights into surface corrosion, internal structures, and composition.

#### 4.4.2 Potential CoMic techniques

**FIGURE 14:**
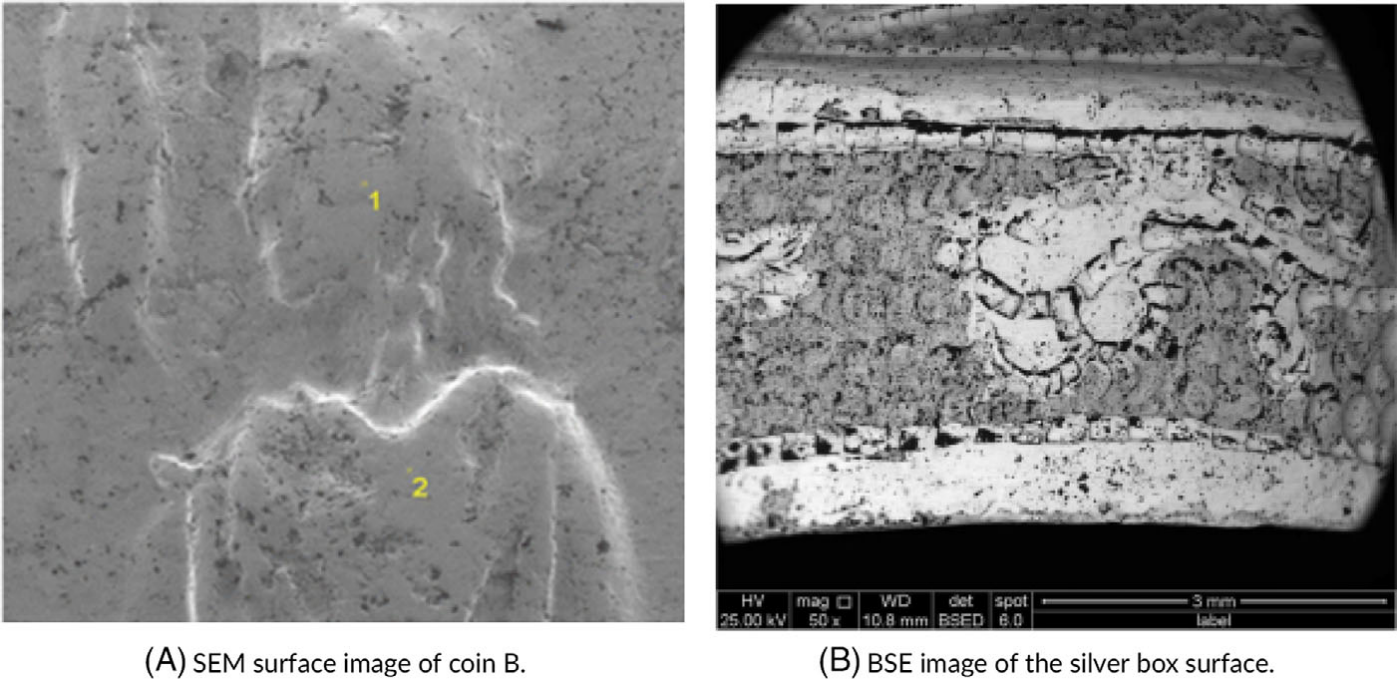
Microscopy and imaging of metal artefacts using SEM and BSE techniques. Reproduced from Refs. 128, 129, licensed under CC BY 4.0.

X‐ray microscopy is particularly valuable in visualising internal features of metal artefacts, detecting voids, cracks, and corrosion layers that are not visible on the surface.^119^ SEM provides high‐resolution imaging of surface textures and enables the analysis of corrosion patterns, which is useful in assessing the artefact's structural integrity.^120^ EDS, often used with SEM, enables elemental composition analysis, helping to identify materials used and any degradation products.^121^ By integrating these methods, CoMic offers a thorough, non‐destructive analysis of metal artefacts, giving conservators critical information for preservation planning. Figure 14 illustrates these applications. Figure 14A shows an SEM surface image of a coin, where surface roughness, pits, and early‐stage corrosion features are visible across the relief of the design. These microstructural defects indicate points where corrosion has initiated, compromising the fine details of the coin's surface. Figure 14B presents a BSE image of the silver box surface, where differences in grey‐level contrast reveal variations in alloy composition and corrosion stratigraphy. Localised bright and dark regions highlight inhomogeneities, voids, and inclusions within the silver, features that both document historical manufacturing practices and serve as markers of ongoing degradation. Together, the SEM and BSE images emphasise how surface and subsurface features (such as bubbles, voids, and corrosion layers) can be identified and linked directly to long‐term preservation risks.

#### 4.4.3 Conservation implications

**FIGURE 15:**
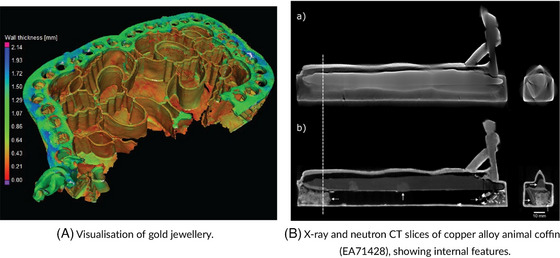
Microscopy and imaging of metal artefacts using CT techniques. Reproduced from Refs. 130, 131, licensed under CC BY 4.0.

The insights gained from CoMic techniques guide conservators in identifying structural weaknesses, understanding corrosion patterns, and making informed decisions about stabilisation treatments. For example, EDS can detect chemical changes in alloy composition due to environmental exposure,^122^ while X‐ray microscopy allows conservators to assess internal voids and cracks.^118^ This integrated approach supports a conservation strategy that preserves the integrity of the artefact while protecting it from further environmental damage. The use of CT techniques to create 3D visualisations of gold jewellery and to inspect the internal features of sealed copper alloy coffins is shown in Figure 15, with Figure 15A showing a wall thickness map of a piece of gold jewellery, colour‐coded to reveal variations in thickness that indicate areas of mechanical vulnerability, providing information on ancient manufacturing methods, including soldering and hollow construction. Information such as this allows conservators to predict where cracks or wear are most likely to occur. In Figure 15B, complementary X‐ray and neutron CT slices through a sealed copper alloy coffin (EA71428) expose otherwise inaccessible internal features, including joints, internal fills, and hidden fractures. This approach enables the study of intact and fragile artefacts without invasive sampling, ensuring that structural and material information can be recovered while preserving the object. Together, the examples in Figures 14 and 15 demonstrate how CoMic‐based approaches (from surface imaging to 3D volumetric mapping) reveal both visible and concealed features, supporting conservation strategies that balance preservation with minimal intervention.

#### 4.4.4 Corrosion

Corrosion product characterisation significantly enhances understanding of metallic artefact degradation. For example, Raman spectroscopy has detected corrosion compounds such as ${\mathrm{Fe}}_{3}O$ 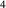, γ‐FeOOH, α‐FeOOH, and α‐${\mathrm{Fe}}_{O}2$ 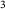 in Chinese iron relics, guiding restoration material selection.^123^ In‐depth Raman and EDS analyses of oxide layers (e.g. magnetite, goethite, lepidocrocite) provide insight into environmental exposure and inform conservation protocol development. Synchrotron‐based methods have further expanded these capabilities. Micro‐XRD and μXANES at synchrotron facilities have been used to characterise poorly crystalline iron corrosion products in archaeological artefacts, revealing transformation pathways within the corrosion system that are difficult to resolve by laboratory methods.^124^, ^125^, ^126^ More recently, synchrotron μXANES has been applied to aluminium alloys from heritage aircraft, enabling phase identification of highly altered, nanocrystalline corrosion layers and informing tailored preservation strategies.^127^ Comparable synchrotron XRD analyses have also been extended to bronze and copper alloy heritage objects, offering insights into corrosion stratigraphy and environmental interactions.^126^ Incorporating such examples underlines the practical relevance of corrosion‐phase identification for informed treatment strategies and highlights how multimodal approaches combining Raman, EDS, and synchrotron techniques can provide a robust picture of long‐term material stability.

### 4.5 Textiles

#### 4.5.1 Material properties and challenges

Textile artefacts, such as ancient tapestries or garments, are among the most vulnerable in heritage collections. They often suffer from fibre degradation, microbial growth, fading, and environmental sensitivity due to their organic composition.^132^ The fragility of natural fibres, like silk or cotton, increases with age, and the presence of dyes and historical treatments can complicate conservation.^133^ Understanding fibre deterioration and chemical composition is essential for developing safe and effective preservation strategies.

#### 4.5.2 Potential CoMic techniques

**FIGURE 16:**
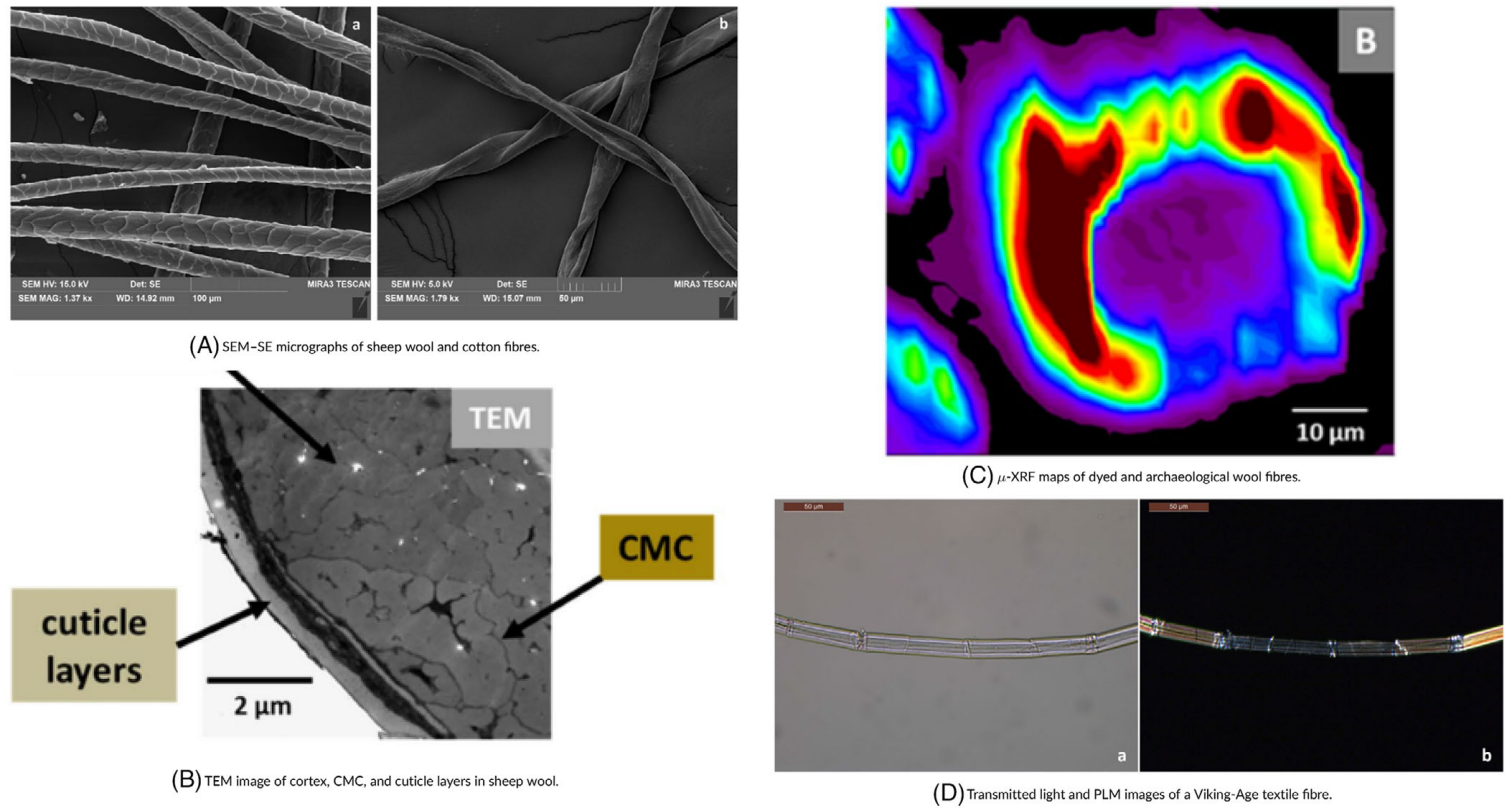
Microscopy and imaging of textile artefacts using SEM, TEM, μ‐XRF, and PLM techniques. Reproduced from Refs. 54, 138, licensed under CC BY 4.0.

SEM is well‐suited for examining fibre morphology, revealing the structure of degraded or brittle fibres.^134^ FTIR spectroscopy and Raman microscopy can analyse organic compounds within the fibres and dyes, providing insight into the chemical composition and identifying any historical treatments or contaminants.^135^, ^136^ AFM can measure fibre elasticity and brittleness, which is valuable in assessing the condition and handling requirements of the textile.^137^ By combining these techniques, CoMic provides a non‐destructive method to assess the material composition, structure, and preservation needs of textile artefacts. Figure 16 displays a range of imaging techniques applied to textile artefacts, including SEM, TEM, μ‐XRF, and PLM, to analyse fibre morphology and composition.

#### 4.5.3 Conservation implications

The application of CoMic techniques allows conservators to identify the types and extent of fibre degradation, determine the presence of microbial contamination, and understand the chemical makeup of dyes and treatments. For example, FTIR can detect organic compounds that signal microbial activity, allowing targeted cleaning interventions. SEM's detailed imagery enables conservators to assess fibre fragility, guiding handling and stabilisation techniques that prevent further damage.

## 5 CHALLENGES AND LIMITATIONS

While CoMic has the potential to revolutionise the study of cultural heritage artefacts, its implementation and interpretation are not without challenges and limitations. This section explores the various hurdles faced in this field, offering a realistic perspective on the current state of CoMic and the areas that necessitate further development. A summary matrix of these practical challenges across the major microscopy categories is provided in Table 2.

**TABLE 2:** Summary matrix listing key practical challenges across major microscopy technique categories.

Technique	Destructive	Costly	Time‐intensive	Needs expertise	Complex prep	Large data
Electron microscopy	✓*	✓	✓	✓	✓	✓***
X‐ray microscopy	✗**	✓†	✓	✓	✓	✓
Optical microscopy	✗	✗	✗	✗	✓	✓
Neutron microscopy		✓†	✓	✓	✓	✓
Atomic force microscopy	✗	✓	✓	✓	✓	✗

**Destructive**: Destroys or significantly alters sample during imaging.

**Costly**: Requires expensive equipment or operational costs.

**Time‐intensive**: Long acquisition and/or preparation times.

**Needs expertise**: Requires specialist training to operate and interpret.

**Complex prep**: Involves demanding or lengthy sample preparation.

**Large data**: Generates large or complex datasets requiring specialised handling.

* SEM may be minimally invasive, while TEM and cryo‐EM require sectioning or sample removal.

** X‐ray microscopy is generally non‐destructive in lab‐based systems but may cause damage in synchrotron‐based applications due to higher dose rates.

† whilst operational costs are high, academic access is often free or subsidised at national facilities.

*** Large datasets arise mainly in advanced modes such as confocal, tile scans, etc. with standard imaging yielding low volumes.

### 5.1 Technical challenges

The primary technical hurdles in applying CoMic to heritage science can be grouped into three main areas. These include the intricate and often destructive nature of sample preparation for irreplaceable artefacts, the inherent instrumentation limitations of various microscopy techniques regarding resolution and field of view, and the significant challenges associated with managing and processing the large, multimodal datasets that are generated

#### 5.1.1 Sample preparation

One of the primary challenges in applying CoMic to cultural heritage studies lies in the complexity of sample preparation. Artefacts often comprise heterogeneous materials, each with unique preservation needs and sensitivities to microscopy techniques. Preparing samples for one microscopy method without compromising their suitability for others, or their overall integrity, requires meticulous planning and execution. For instance, techniques like TEM necessitate ultra‐thin sections,^139^ which can be challenging to produce from brittle or composite materials without causing damage. Similarly, SEM and certain volume EM methods require conductive coatings that might not be reversible, posing a risk to precious artefacts.^140^ Moreover, the need to maintain the original context and provenance of artefacts further complicates sample preparation, as any alteration could lead to loss of valuable historical information. Current research is addressing these challenges through the development of innovative, less invasive sample preparation techniques. Advances in cryo‐preparation methods allow for the preservation of hydrated states and delicate structures, reducing artefact alteration risks.^141^ Additionally, the use of non‐conductive coatings for SEM and the exploration of low‐voltage electron microscopy are promising solutions for minimising sample damage.^141^ Researchers are also leveraging micro‐sampling techniques, ensuring minimal intervention while still obtaining sufficient material for analysis.^142^ Furthermore, interdisciplinary collaboration between scientists, conservators, and historians is crucial in devising sample preparation strategies that balance analytical needs with conservation ethics. By integrating expertise from various fields, the cultural heritage community is continually refining sample preparation methodologies to overcome these complexities, ensuring artefacts' longevity and historical integrity are preserved for future generations.

#### 5.1.2 Instrumentation limitations

Instrumentation limitations present significant challenges in the application of CoMic to cultural heritage studies. These limitations often stem from the inherent constraints of microscopy techniques, including resolution limits, depth of field, and the ability to analyse specific types of materials or artefacts. For example, the resolution of SEM may not be sufficient for certain nanoscale investigations crucial for understanding fine material details.^143^ TEM, while offering higher resolution, is limited by its requirement for thin sample sections, making it unsuitable for examining the bulk properties of artefacts.^139^ Additionally, techniques like X‐ray microscopy can offer non‐destructive analysis but may lack the resolution needed for detailed surface examinations or may not be sensitive to all elements present in complex artefacts.^144^ The field of view is another critical limitation; some high‐resolution techniques can only examine small areas at a time, making it challenging to get a comprehensive view of larger artefacts. This can lead to a fragmented understanding of the artefact as a whole, potentially missing crucial information about its condition and history. Current research is addressing these instrumentation limitations through technological advancements and methodological innovations. Developments in detector technology and imaging software are continuously expanding the capabilities of existing microscopy techniques, improving resolution, and broadening the field of view.^145^ For instance, advancements in SEM technology, such as the incorporation of FIB for site‐specific milling, have enhanced its application range, allowing for detailed 3D reconstructions of artefact microstructures.^138^ Moreover, the integration of complementary techniques within the CoMic framework helps to mitigate individual instrumentation limitations. By correlating data from multiple microscopy methods, researchers can compile a more comprehensive understanding of artefacts, leveraging the strengths of each technique to overcome their individual weaknesses.

#### 5.1.3 Data management and processing

The integration of CoMic in cultural heritage studies generates vast amounts of complex data, spanning various scales and modalities. This abundance of information presents significant challenges in data management and processing, requiring sophisticated strategies for storage, integration, and analysis. One of the primary issues is the heterogeneity of the data, with different microscopy techniques producing diverse types of information, from high‐resolution images to elemental composition maps and 3D reconstructions. Each data type may require distinct processing algorithms and software, complicating the task of integrating and correlating findings across modalities. Furthermore, the sheer volume of data, particularly from high‐resolution and 3D imaging techniques, demands substantial computational resources for storage and processing. This can pose a barrier for institutions with limited IT infrastructure, potentially restricting access to CoMic's full capabilities.^45^, ^146^ Addressing these challenges involves both technological solutions and methodological innovations. On the technological front, advancements in data storage technologies, such as cloud‐based solutions, offer scalable and accessible options for managing large datasets. High‐performance computing (HPC) environments and distributed computing networks can provide the necessary processing power to handle complex data analyses.^147^ From a methodological perspective, the development of integrated software platforms capable of handling diverse data types from different microscopy techniques is crucial. These platforms should offer user‐friendly interfaces and automated processing workflows to facilitate data integration and correlation, making advanced analyses more accessible to researchers from various disciplines. Efforts to standardise data formats and metadata across microscopy techniques further streamline data integration, enabling more effective collaboration and sharing of findings within the cultural heritage research community. AI and machine learning are increasingly applied in heritage science, particularly for classification, damage detection, and predictive modelling. For example, convolutional neural networks (CNNs) and object‐detection architectures (e.g., YOLOv8) have been trained to identify wall‐surface damage in historic structures, achieving robust performance (F1‐scores ≈0.7 for stain detection).^148^ Corrosion‐related prediction is another emerging application: machine learning models have been used to forecast corrosion rates in stainless steel under varying environmental conditions ^149^ and to design corrosion‐resistant high‐entropy alloys using physics‐informed algorithms.^150^ Beyond structural materials, neural‐network‐based classification of ceramic thin sections has demonstrated >90% accuracy while retaining model interpretability,^151^ offering a scalable means of petrographic analysis in archaeology. Similarly, spectral unmixing approaches driven by machine learning have improved pigment identification in heritage paintings and manuscripts.^152^ Together, these developments illustrate how AI can enable efficient diagnostic workflows, predictive conservation strategies, and enhanced interpretability in heritage studies. Importantly, coupling AI with multimodal microscopy (e.g., Raman, XRF, XRD‐CT) provides a route toward integrated data analysis pipelines that move beyond descriptive imaging, towards predictive modelling of artefact condition and long‐term stability. By addressing these data management and processing challenges, CoMic can unlock new insights into cultural heritage artefacts, fostering a deeper understanding of our shared history and heritage. The application of artificial intelligence (AI) and machine learning (ML) is becoming increasingly important in addressing these challenges. Recent studies have demonstrated their use in heritage science for classification, damage detection, and predictive modelling.^153^, ^154^ By enabling automated data integration and improved interpretability, AI and ML approaches offer promising solutions for managing the large and complex datasets generated by CoMic workflows.

### 5.2 Methodological challenges

Beyond the technical aspects of the instruments, significant methodological challenges also exist. These primarily concern the difficulty in achieving accurate data correlation between different imaging modalities and the widespread lack of standardised procedures, which can impede the reproducibility and comparison of results across different research institutions.

#### 5.2.1 Correlation accuracy

Achieving accurate data correlation is a central challenge in CoMic, particularly when combining different modalities such as SEM, X‐ray CT, and optical microscopy. Variations in resolution, imaging depth, and data type can complicate the alignment of datasets, potentially leading to inaccurate conclusions about the condition or composition of artefacts. For instance, aligning surface data from SEM with subsurface information from X‐ray CT requires precise calibration to ensure that the combined datasets reflect the true physical structure of the artefact.^155^ To address this, researchers are refining image registration techniques and developing advanced software tools that facilitate more accurate data alignment.^156^ The use of fiducial markers‐visible across multiple microscopy platforms‐helps improve spatial correlation. Ongoing developments in automation aim to minimise human error and enhance the precision of these correlative analyses, contributing to a more reliable interpretation of artefact features.

#### 5.2.2 Standardisation of procedures

The lack of standardisation in CoMic methods presents challenges in ensuring reproducibility and comparability of results across different research institutions. As discussed in Section 5.1.1, sample preparation remains a critical challenge, particularly in ensuring artefact integrity across multiple modalities. Variability in image conditions and data analysis can hinder efforts to aggregate the findings of multiple studies^146^, ^157^. To overcome this, interdisciplinary working groups would be useful to develop guidelines for handling artefacts, setting imaging parameters, and reporting data. These efforts could create common standards that cater to the unique needs of cultural heritage studies. In parallel, open‐access platforms are facilitating the sharing of standardised protocols and datasets, promoting transparency and collaboration.^155^, ^156^ Training workshops and educational initiatives could further support the standardisation process by ensuring that researchers and conservators adhere to established protocols, fostering consistency across the field.

### 5.3 Resource and accessibility issues

Practical barriers related to resources and accessibility also limit the widespread adoption of CoMic in cultural heritage research. These issues include the high costs associated with the initial investment, maintenance, and operation of advanced microscopy systems, as well as the need for highly specialised, interdisciplinary expertise to properly operate the equipment and interpret the data.

#### 5.3.1 High costs and resource intensity

The financial and resource demands of CoMic remain significant barriers to its widespread adoption in cultural heritage research. Advanced microscopy systems, such as SEM and volume EM methods, require substantial capital investment, ongoing maintenance, and skilled personnel for operation.^146^, ^157^ Additionally, the computational infrastructure needed to process and store the large datasets generated by CoMic adds further costs. Collaborative frameworks, such as shared facility models, offer a potential solution. By pooling resources, multiple institutions can access high‐end microscopy equipment without bearing the full financial burden. Grant funding, public‐private partnerships, and the development of more cost‐effective microscopy technologies also play key roles in making CoMic more accessible. Open‐source software for data processing is another avenue being explored to reduce costs while maintaining high‐quality analysis capabilities.^158^

#### 5.3.2 Requirement for specialised expertise

The successful implementation of CoMic requires specialised expertise that spans disciplines, from materials science and chemistry to conservation and art history,^12^, ^83^, ^157^ and exemplified by the diverse user profiles outlined in Table 1. This interdisciplinary knowledge is essential not only for operating complex microscopy equipment but also for interpreting the data in the context of an artefact's historical and cultural significance.^31^, ^146^ Educational initiatives, such as specialised university courses and professional workshops, are crucial in addressing this expertise gap. Hands‐on training programmes and interdisciplinary collaborations further support the development of skilled professionals who can effectively apply CoMic techniques to cultural heritage studies.

### 5.4 Ethical and conservation considerations

Finally, the application of CoMic in this field is governed by crucial ethical and conservation considerations. These encompass the overriding priority for non‐destructive analysis to ensure the preservation of artefacts for future generations, alongside the need for deep cultural sensitivity when studying objects that hold significant cultural or religious value.

#### 5.4.1 Non‐destructive analysis

In cultural heritage, the need for non‐destructive analysis is paramount to ensuring the preservation of artefacts for future generations. While many microscopy techniques are inherently non‐invasive, others – such as TEM, which requires thin sectioning – pose risks to the integrity of artefacts. Advances in X‐ray microscopy, surface scanning, and Cryo‐EM are providing alternatives that allow for detailed analysis without compromising the artefact's physical state.^16^, ^159^ As CoMic methodologies evolve, a careful balance must be struck between the information gained and the potential risks to artefact preservation. In many cases, non‐destructive techniques are used first to assess the feasibility of more invasive methods, ensuring that artefact integrity is always prioritised.

#### 5.4.2 Cultural sensitivity

Cultural sensitivity is a critical consideration in CoMic applications, particularly when working with artefacts that hold significant cultural or religious value. Close collaboration with cultural stakeholders – such as indigenous communities, historians, and cultural custodians – is essential to ensuring that research respects the artefacts' cultural significance.^16^, ^160^ This approach fosters transparency and respect, allowing for a more holistic understanding of artefacts that includes traditional knowledge and cultural perspectives. Guidelines that prioritise cultural sensitivity are being developed and disseminated by professional organisations, ensuring that CoMic research is conducted ethically and respectfully. By integrating scientific and cultural approaches, researchers can gain deeper insights while preserving the artefacts' cultural heritage.

## 6 CONCLUSIONS AND FUTURE PERSPECTIVES

**FIGURE 17:**
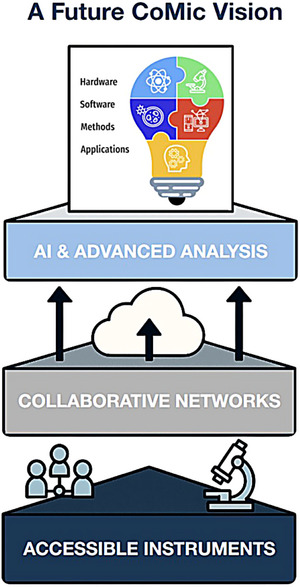
Future vision for CoMic in cultural heritage: integration of AI‐driven analysis, cloud‐based data handling, accessible lab‐based instrumentation, and collaborative research networks to support predictive, reproducible, and widely accessible workflows.

CoMic has emerged as a transformative approach in cultural heritage studies, enabling non‐invasive, multimodal analyses that provide unprecedented insights into the structure, composition, and condition of artefacts. This review has highlighted key methodological advances and case studies, while also identifying persistent challenges such as correlation accuracy, standardisation, and resource accessibility. Looking ahead, progress will depend on both technical innovation and collaborative practice. Advances in high‐resolution, non‐destructive imaging and the wider availability of cost‐effective instrumentation will extend the reach of CoMic to under‐resourced institutions. Interdisciplinary collaboration will be central to addressing data management challenges, particularly through the integration of artificial intelligence and machine learning for automated processing, predictive modelling, and improved interpretability. Efforts to establish shared protocols and interoperable data standards will be critical for enabling reproducible workflows and facilitating effective data exchange across the cultural heritage community. Such standardisation will enhance comparability of results while supporting collaborative research at national and international levels. Future applications are expected to expand to a broader range of materials, including low‐density and organic artefacts, while digital reconstructions and simulation‐based approaches may provide a route to engage with intangible heritage. By combining emerging technologies with cross‐disciplinary expertise, CoMic has the potential to drive predictive conservation strategies, enrich historical interpretation, and ensure the protection of cultural treasures for generations to come. A model for this future vision, based on a foundation of accessible instruments, collaborative networks, and advanced AI‐driven analysis, is illustrated in Figure 17.

## CONFLICT OF INTEREST STATEMENT

The authors declare that they have no known competing financial interests or personal relationships that could have appeared to influence the work reported in this paper.
